# Oscillatory network efficiency predicts mood and fatigue during sleep deprivation

**DOI:** 10.1038/s42003-026-10250-8

**Published:** 2026-05-16

**Authors:** David Negelspach, Alisa Huskey, Kathryn E. R. Kennedy, Jungwon Cha, Michael A. Grandner, Daniel Forger, William D. S. Killgore

**Affiliations:** 1 Department of Psychiatry, Social, Cognitive, and Affective Neuroscience Lab, College of Medicine, University of Arizona, Tucson, AZ USA; 2 Department of Psychiatry, Sleep and Health Research Program, College of Medicine, University of Arizona, Tucson, AZ USA; 3 Department of Mathematics, University of Michigan, Ann Arbor, MI USA

**Keywords:** Neuroscience, Sleep deprivation

## Abstract

Fluctuations in performance and mood across the day have been traced to circadian and homeostatic modulation of motor and affective systems, although their combined influence on network topology is rarely considered. We applied a data-driven curve-fitting algorithm to capture both circadian and infradian rhythms ( ≥ 24 hours) in frontolimbic and sensorimotor regions using functional magnetic resonance imaging (fMRI) measures of connectivity. Across the course of sleep deprivation, functional network structure was not static but changed in tandem with objective and subjective behavioral measures. Oscillatory patterns in network efficiency suggest that circadian rhythmicity extends to higher-order network topology. Sleep deprivation affects functional networks in a region-specific manner, highlighting local vulnerability. Distinct cortical regions exhibited unique circadian phases of network reorganization, revealing that connectivity rhythms are spatially as well as temporally differentiated across the brain. Time-dependent alterations in connectome topology offer a systems-level framework for understanding how internal timekeeping and sleep pressure modulate non-linear trends in psychomotor vigilance, mood, and fatigue across extended wakefulness.

## Abstract (web summary)

Efficiency of regional networks in frontal, limbic, and sensorimotor regions show unique oscillatory patterns of reorganization. Variation in psychomotor vigilance, fatigue, and negative affect reflect dynamic changes in functional network topology.

## Introduction

Throughout the course of the day, human behavior and cognitive ability are simultaneously influenced by circadian phase and homeostatic sleep pressure. Factors such as time awake and chronotype contribute to an oscillatory pattern of performance in cognitive tasks requiring attention, working memory, and decision making (for review see ref. ^1^). The underlying neurobiological rhythms that accompany these performance trends are adaptive when experienced during typical waking hours. However, prolonged or improperly timed wakefulness can render these rhythms maladaptive, leading to heightened emotional reactivity and impaired decision-making^2^. Under such conditions, circadian-phase-driven changes in brain network connectivity may exacerbate the cognitive deficits of sleep loss, resulting in periods of exaggerated emotional vulnerability and diminished self-regulatory control.

Endogenous circadian rhythms orchestrate fluctuations in physiology and behavior over the 24-h day, including subtle but measurable oscillations in brain activity. Neuroimaging studies have shown that resting-state network connectivity exhibits time-of-day–dependent variation^3,4^. Similar studies show that diurnal changes in functional connectivity vary with chronotype, further indicating that circadian phase affects large-scale network organization of the brain^5,6^. These findings suggest that the functional architecture of the brain is not static throughout the day but reorganizes in a manner that is influenced by circadian processes. Such dynamic changes in connectivity may contribute to circadian components of cognitive functioning such as learning and memory^7^ and psychomotor vigilance^8^. Extending this line of reasoning, it is also plausible that neural network reorganization associated with circadian phase may also influence other cognitive domains, including the processing of emotional stimuli.

Emotional regulation, like other cognitive functions, shows signs of diurnal variability with circadian modulation. This relationship is evident in research showing that circadian misalignment is frequently associated with deficits in emotional regulation^9^. While a causal role cannot be inferred from co-morbidity, more direct experimental approaches inducing circadian desynchrony through simulated shift work have been shown to enhance depressive mood^10^. Different chronotypes also show significant differences in emotional state; evening type individuals tend to report higher levels of depressive mood when compared to morning types^11^. This evidence suggests that circadian fluctuations may not only affect arousal, but also have an impact on circadian patterns of positive and negative affect. Extending wakefulness into the biological night may heighten emotional reactivity, in part due to altered functional dynamics driven by circadian phase.

In addition to circadian influences on emotional state, sleep loss impairs emotional regulation through the accumulation of homeostatic sleep pressure and associated disruptions in prefrontal–limbic balance. Individuals experiencing insufficient sleep often display heightened emotional reactivity, particularly in response to challenging or negative stimuli^12,13^. These behavioral effects are thought to reflect disrupted top-down control of limbic circuits, especially via the prefrontal cortex^14^. For example, heightened emotional reactivity following short-term sleep loss has been linked to impaired functional connectivity of the prefrontal cortex^15^ and increased amygdala activation^16^. This frontal vulnerability under sleep deprivation has been described as a state of disinhibition, whereby impaired prefrontal control permits exaggerated limbic responses^17^. Together with circadian misalignment, frontolimbic vulnerability to sleep deprivation may magnify emotional instability.

The combined influence of circadian and homeostatic factors on emotional deficits during sleep deprivation make the timing of functional imaging or behavioral assessment a critical experimental consideration. This is because the neurobiological effects of sleep deprivation are not entirely independent of circadian phase. Circadian timekeeping can affect homeostatic sleep pressure and vice versa^18^. For example, deficits in working memory and psychomotor vigilance can be transiently improved during the wake maintenance zone despite sleep deprivation^19,20^. Thus, circadian influences on functional properties are superimposed on, and sometimes mask, underlying homeostatic declines in performance. It remains unclear whether circadian influences on brain function are limited to the wake-maintenance zone or act continuously throughout the day. Many neuroimaging studies adopt a binary-contrast design—comparing a rested baseline to a maximally sleep-deprived state—which obscures temporal dynamics. This is particularly consequential for emotion-related circuits, since impaired mood regulation is a well-documented consequence of sleep loss. Mapping how prefrontal–limbic networks evolve in response to sleep-loss and circadian cycles could reveal windows of resilience and vulnerability, with important mental-health implications given the frequent comorbidity of affective disorders and sleep disturbance^21^.

Modeling how functional network architecture changes throughout a period of sleep deprivation requires an interactive consideration of homeostatic and circadian processes. However, there are few readily available analytical frameworks capable of modeling the interdependent dynamics of circadian phase and sleep pressure. Traditional circadian models, such as cosinor analysis, typically require an initial period assumption and is not well-suited to capture multiprocess interactions^22^. Data-driven methods for determining optimal data fit without a-prior assumptions would be preferred. The current study addresses these challenges by applying a curve-fitting algorithmic approach to model circadian and homeostatic influences on functional measures. We hypothesize that sensorimotor and frontolimbic networks dynamically reconfigure under the joint influence of circadian phase and homeostatic sleep pressure, producing transient topological states that mediate the cognitive and affective deficits of sleep deprivation.

## Results

### Descriptive behavioral data

**Fig. 1:**
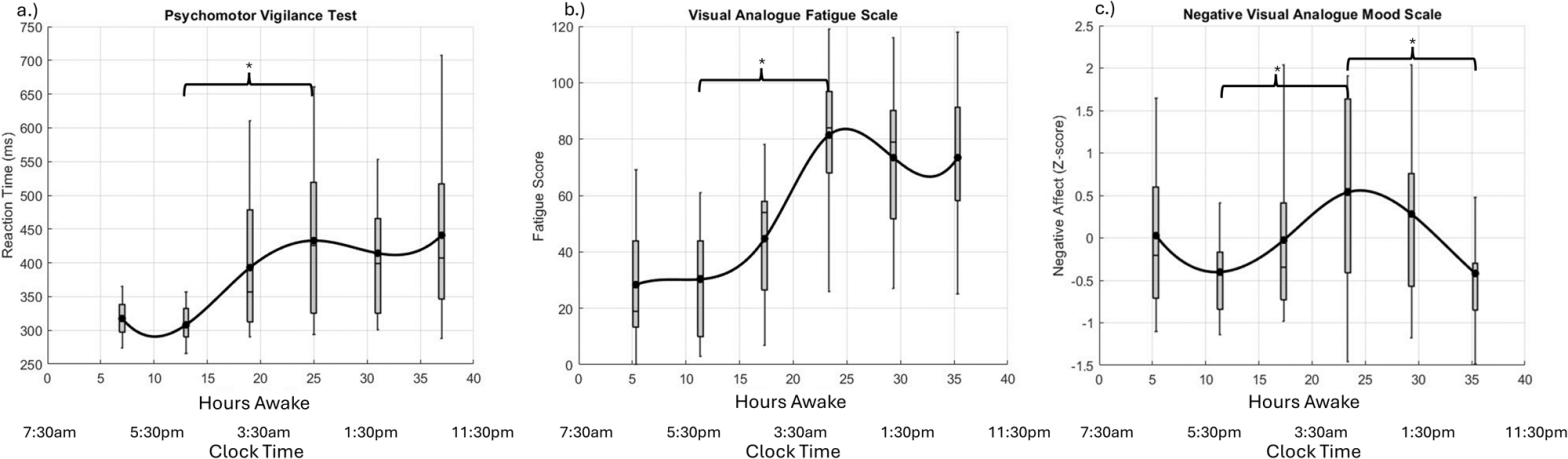
Repeated assessments of vigilance, fatigue, and negative mood measured across sleep deprivation. **a**–**c** Behavioral measures were collected at six timepoints spaced six hours apart over a 39-h sleep deprivation protocol. Each point represents the group mean. Box-and-whisker plots are overlaid at each timepoint showing the median, interquartile interval, and range. Within subject comparisons were conducted at 12-h intervals beginning on the first night, with significant changes indicated with an asterisk (*p* < 0.05). Negative affect scores reflect a subcomponent of the VAMS and were within-subject normalized (Z-scored) to account for individual variability in baseline state and emotional vulnerability.

Group-aggregated behavioral measures are shown at six-hour intervals throughout a period of sleep deprivation: PVT (Fig. 1a), VASF (Fig. 1b), and negative VAMS (Fig. 1c). Within-subject t-tests were used to compare behavioral scores across 12-hour intervals to assess opposing circadian phases and homeostatic effects. As expected, extending wakefulness into the night impaired psychomotor vigilance, and progressively increased subjective fatigue (Table 1). Subsequent responses on the VASF and PVT remained significantly impaired but did not show any additional significant deficits in the 12-h intervals.

**Table 1:** Behavioral changes across 12-h sleep deprivation intervals

Behavioral variable	Timepoint (T) comparison	Statistic (degrees of freedom)	p	95% Confidence interval (CI)	Cohen’s d
PVT	T2–T4	t(18) = 5.32	4.63e-5*	[75.54, 174.02]	1.22
	T4–T6	T(18) = 0.33	0.75	[-43.49, 59.45]	0.07
VASF	T2–T4	T(17) = 8.42	1.82e-7*	[38.71, 64.62]	1.98
	T4–T6	T(18) = −1.72	0.10	[−17.79, 1.79]	−0.39
VAMS	T2–T4	T(17) = 3.48	2.85e-3*	[0.37, 1.51]	0.82
	T4–T6	T(17) = −3.01	7.81e-3*	[−1.62, -0.29]	−0.71

Accompanying statistics for within subject comparisons of behavioral variables. Timepoint comparisons were conducted at 12-hour intervals starting from the first night. For each comparison, the t-statistic, degrees of freedom, *p*-value, 95% confidence interval (CI) of the mean difference, and effect size (Cohen’s d) are reported.

Changes in negative affect were highly variable and showed evidence of oscillation (Fig. 1c). Subjective negative components on the VAMS also significantly increased past the typical time awake, reaching a maximum at approximately 6:00 am. Self-reported negative affect decreased significantly thereafter between 23 and 35 h awake.

### Cosinor-analysis of graph theory measures

**Fig. 2:**
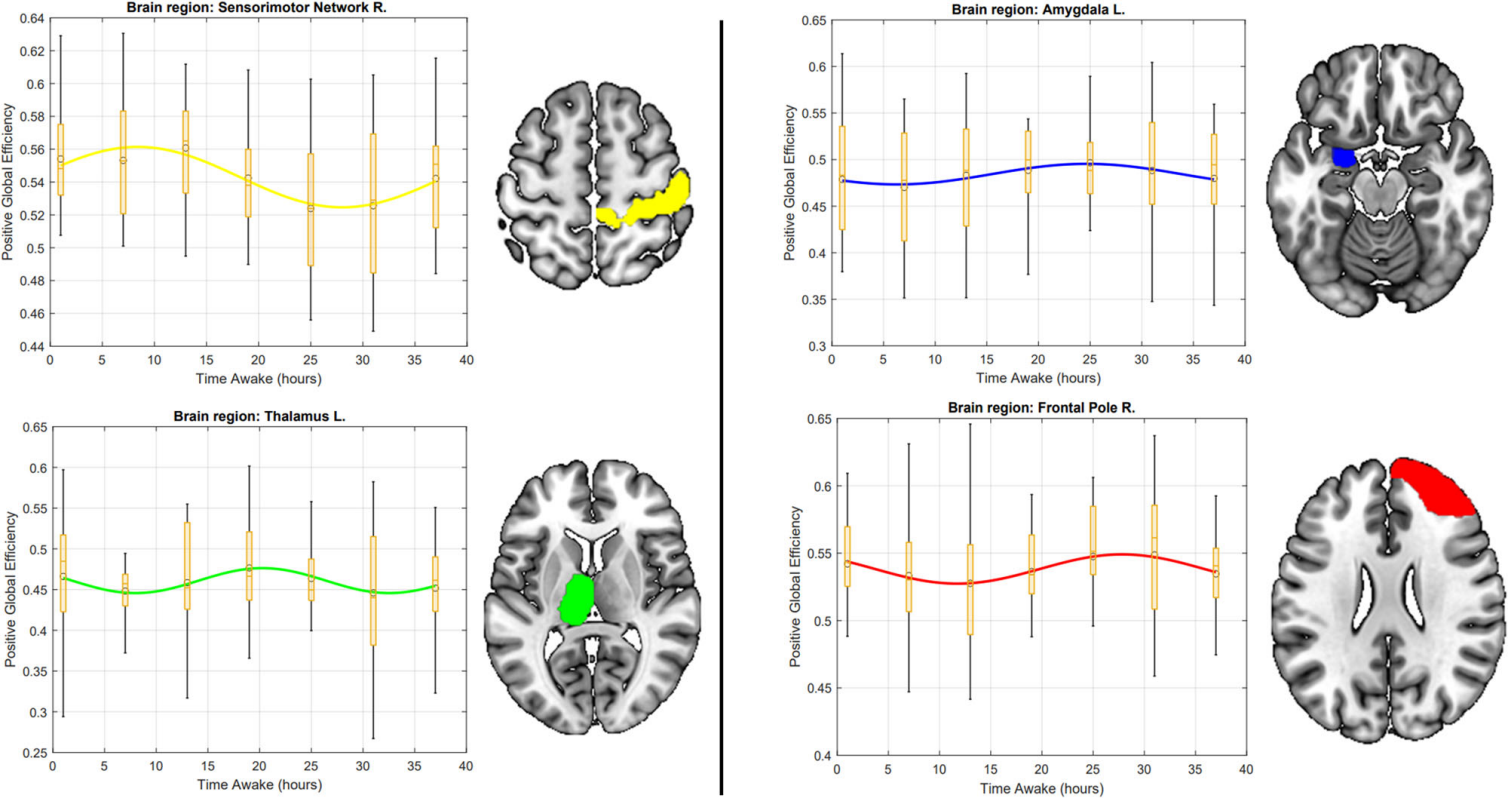
Each graph shows the fitted cosinor function applied to calculations of regional network efficiency for each predefined ROI. Representative axial slices are shown adjacent to each line graph, corresponding to the ROI in which regional efficiency values were calculated. Z-coordinates for each axial slice are as follows: postcentral gyrus (*z* = 58), frontal pole (*z* = 25), thalamus (*z* = 8), and amygdala (*z* = –17). False discovery rate correction was applied *p* < 0.05. Individual voxel thresholds were maintained at the standard *p * <  0.001. Predefined anatomical ROIs and line graphs are color-coded. Box-and-whisker plots are overlaid at each timepoint showing the median, interquartile interval, and range. R right, L left.

Global efficiency measures extracted from anatomically defined ROIs were modeled using a cosinor-based curve-fitting algorithm (Fig. 2). Across sleep deprivation, significant oscillatory patterns of global efficiency emerged in four anatomically defined ROIs: right frontal pole, left thalamus, left amygdala, and right postcentral gyrus. Each region exhibited a distinct phase, period, and amplitude, indicating region-specific functional reorganization over time. Notably, the thalamus demonstrated the most circadian pattern, whereas other regions maintained oscillatory periods longer than 24-h. Fitted parameters and statistics of each optimized cosinor function for each region are shown in Table 2.

**Table 2:** Fitted Cosinor parameters in the frontal, limbic, and sensorimotor regions

Anatomic region	Cluster centroid	F(3,3)	p	Midline	|Amplitude| [95% CI]	Period (h)
R. Frontal Pole	(27, 52,9)	39.32	0.00658	0.538	0.0108 [0.0091, 0.0125]	32.17
L. Thalamus	(−10,−18,7)	24.55	0.0129	0.461	0.0153 [0.0123, 0.0184]	24.55
L. Amygdala	(−24,−1,−18)	12.85	0.0322	0.484	0.011 [0.008, 0.0142]	35.76
R. Postcentral Gyrus	(38,−26,52)	10.15	0.0444	0.543	0.0184 [0.0124, 0.0242]	39.27

Results from algorithmic analysis of ROI global efficiency are shown. Anatomical regions corresponding to each ROIs is listed along with the Montral Neurological Institute coordinates of each cluster centroid. F-statistics and corresponding *p* values are shown for each fitted curve with respect to the raw data. Fitted oscillatory parameters for each function, including the fitted period, and midline. Rhythm magnitude is quantified using the estimated amplitude and its 95% confidence interval (CI).

### Maximum changes in frontolimbic network efficiency states

**Fig. 3:**
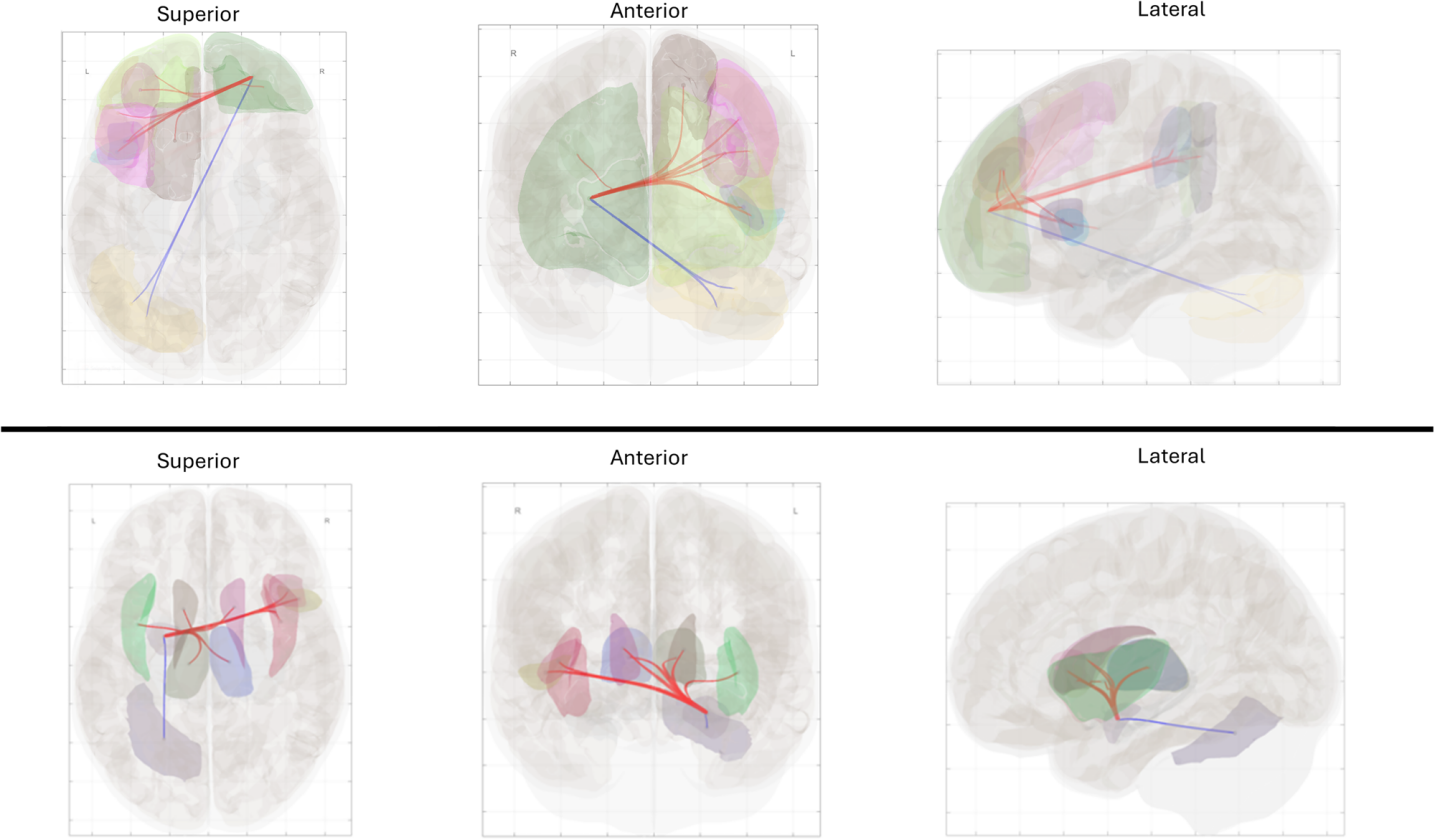
Changes in ROI-ROI connectivity for the right frontal and left amygdala regions are projected onto a glass brain. Red lines indicate significantly increased connectivity, whereas blue lines represent significant decreases in connectivity that occurred over the course of sleep deprivation. Contrasts were conducted between the local maxima and minima of regional global efficiency to extract dominant changes in connectivity patterns. Colors of each ROI were randomly assigned for visual differentiation. All changes in connectivity are significant after false discovery cluster correction p < 0.05 using a parametric random field theory framework.

To quantify specific changes within prefrontal and amygdala networks, changes in functional connectivity were determined between timepoints of maximum and minimum network efficiency (Fig. 3). As oscillation phases differed between regions, the timepoints of maximum and minimum global efficiency were region-specific. Across subjects, peak-to-trough network efficiency occurred between 13 and 31 h of wakefulness for the right frontal pole and between 6 and 25 h of wakefulness for the left amygdala. Heightened amygdala network efficiency coincided with significantly stronger bilateral connectivity with the thalamus, caudate, insula, and right frontal opercular cortex (p_fdr_ < 0.05). A significant decrease in connectivity also occurred between the left amygdala and left cerebellum (region VI). In the right frontal pole, peak-to-trough contrasts revealed strengthened connections with the salience network and prefrontal cortex. Specifically, increased connectivity was observed with salience network regions including the bilateral rostral prefrontal cortex, the left anterior insula, as well as prefrontal regions such as the left frontal pole, left superior frontal gyrus, left middle frontal gyrus, left lateral prefrontal cortex, and left frontal operculum (p_fdr_ < 0.05). Decreased connectivity was also observed between the right frontal pole and left cerebellar lobules (I & II). Changes in connectivity are provided in the Supplementary Data 1. Timepoint-by-timepoint changes in frontal and amygdala networks are shown in Supplementary Fig. 1.

### Subject-level mixed linear modeling

**Fig. 4:**
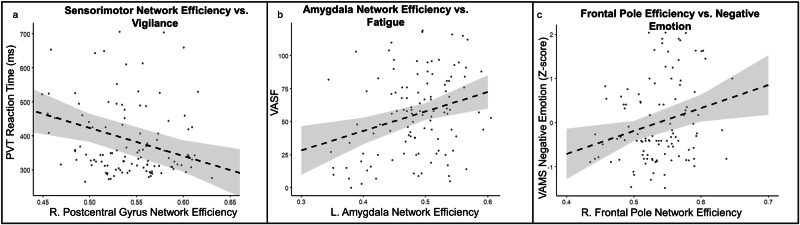
Linear Mixed-Effects Modeling of Regional Connectivity and Behavior. **a** Mixed linear model with scatterplot showing the relationship between PVT reaction time and Postcentral gyrus global efficiency. **b** Mixed linear model with scatterplot showing the relationship between VASF fatigue ratings and Amygdala global efficiency. **c** Mixed linear model with scatterplot showing the relationship between negative VAMS scores and Frontal Pole global efficiency. Each point represents a single observation from individual subjects. The dashed black line shows the predicted population-level relationship from the linear mixed-effects model. The shaded area shows the 95% confidence interval representing the uncertainty in the fixed effect prediction. Statistical models included subject as a random intercept to account for individual variability.

To further explore whether changes in network efficiency predicted behavioral outcomes, a mixed linear modeling approach was applied. In these models, random effects were included across participants, enabling a direct analysis of the association between network efficiency and behavioral outcomes, while accounting for differences in baseline performance (Fig. 4). First, a mixed linear model was calculated for PVT and network connectivity of the postcentral gyrus, which is a key node of the sensorimotor network involved in psychomotor vigilance^23,24^. Global efficiency within the postcentral gyrus significantly predicted PVT reaction times across the period of sleep deprivation (Fig. 4a; *b* = −827.09, 95% CI [−1316.14, −338.03], t(93) = −3.36, *p* = 0.0011). Next, connectivity measures from the amygdala were used to predict subjective fatigue ratings (VASF), based on previous research linking amygdala connectivity to fatigue^25^. Global efficiency within the amygdala significantly predicted subjective fatigue levels (Fig. 4b; *b* = 147.43, 95% CI [52.53, 242.32], *t*(90) = 3.09, *p* = 0.0027). Finally, we examined the relationship between global efficiency in the frontal pole and subjective negative emotional evaluation, as measured by the VAMS. Mixed linear modeling confirmed a significant association between global efficiency in the frontal pole and negative emotional components (Fig. 4c; *b* = 5.20, 95% CI [1.20, 9.20], t(88) = 2.58, *p* = 0.011).

## Discussion

Understanding how biological rhythms influence large-scale brain networks is a central question in chronobiology and cognitive neuroscience. While several behavioral rhythms—such as fluctuations in emotional state—exhibit oscillatory patterns, it remains unclear whether these rhythms correspond to reorganization in network architecture. Analyzing global efficiency provides a systems-level perspective of how information integration and segregation evolve, capturing functional reorganization that raw connection strengths alone cannot reveal. Using a curve-fitting method, both circadian and infradian rhythms in network efficiency, and thus functional topology, were identified in both the frontolimbic and sensorimotor regions. Mixed linear modeling revealed these oscillations to be behaviorally relevant, as they predicted individual variations in mood, fatigue, and vigilant attention. Corresponding changes in connectivity within the right prefrontal and left amygdala regions highlight specific network interactions in accordance with altered emotional processing. Together, these results support our hypothesis that homeostatic and circadian factors drive changes in network topology and are associated with subjective and objective deficits associated with sleep deprivation.

Accumulating evidence indicates that the thalamus is strongly modulated by circadian signals, showing rhythmic changes in connectivity and excitability that influence cortical gating. Animal research shows that subthalamic nuclei demonstrate highly rhythmic clock-gene activity^26^ and profiles of neural activity potentially regulating sleep-wake transitions^27^. In humans, the thalamus also displays circadian patterns of task-based activation that closely parallel melatonin secretion profiles^28^. Thalamic connectivity reveals a similar pattern where sleep deprivation elicits a pronounced breakdown in attention^29^ and cortical connectivity following the wake maintenance zone^30^. Importantly, prior work has established that thalamic activity plays a central role in regulating arousal and sensorimotor responsiveness. Portas et al. demonstrated that the thalamus mediates interactions between attention and arousal state in humans^31^, while Chee and colleagues (2008; 2010) showed that sleep deprivation alters thalamic activation during vigilance tasks, particularly related to lapses^32,33^. Our findings, in conjunction with prior work, indicated thalamic contributions to attention and arousal-related responses vary as a function of circadian phase. This pattern is characterized by dynamic changes in thalamocortical network connectivity and topological efficiency, suggesting that the thalamus plays an active role in supporting cortical responsiveness across the day. Such phase-dependent modulation likely contributes to diurnal fluctuations in attentional stability and behavioral alertness.

Endogenous neural rhythms need not be confined to a 24-h cycle. Interactive effects can occur between both circadian and homeostatic processes^18,34–36^, potentially obscuring oscillatory dynamics. Rather than acting independently, circadian and homeostatic effects can be super-imposed on one-another, shaping fluctuations in neural activity^37–39^. While experimental protocols have been developed to isolate the underlying rhythm of interest, circadian contributions to neural oscillations are not so easily disentangled. For example, homeostatic metabolites such as adenosine affects neuronal excitability^40,41^, potentially masking or obscuring emergent circadian effects on cortical regions and functional networks. From a modeling perspective, fitting canonical functions to such data would result in spatial transforms reflecting these interactive effects, which may not be entirely circadian. Curve-fitting algorithms are an effective method to detect oscillatory dynamics that may deviate from canonical patterns, without strict assumptions of period.

In this context, infradian rhythms in regional network efficiency were observed in prefrontal, limbic, and sensorimotor areas, with fitted periods between 30 and 40 h. This timescale may reflect adaptive modulation of emotional and sensorimotor processing, as oscillations significantly predicted individual variability in VAMS, VASF, and PVT performance. Although increased network efficiency is often interpreted as optimized information transfer, it does not always correspond to desired outcomes. Enhanced efficiency can similarly represent a breakdown of spatial network resolution or increased local correlation reminiscent of local sleep^42,43^.

Whereas increased efficiency in the postcentral gyrus predicted better PVT performance, greater efficiency in the right prefrontal cortex and left amygdala was associated with heightened negative affect and subjective fatigue. This pattern likely reflects overactive emotional processing driven by increased network integration (see below). Although such effects may be maladaptive in modern society, this effect is consistent with an evolutionarily conserved mechanism that prioritizes threat detection when cognitive control is reduced^44^.

To further investigate network configurations associated with maximum changes in frontolimbic efficiency, ROI–ROI connectivity was examined. These changes primarily reflected increased interconnectivity within, and between, central nodes of the salience and frontoparietal networks. The salience network is thought to detect behaviorally relevant or interoceptive stimuli, prioritizing further information processing to the appropriate network^45,46^. The frontoparietal network, on the other hand, is crucial for executive functions, including goal-directed cognition^47^. Altered connectivity between salience and prefrontal circuits have repeatedly been associated with impaired emotional processing^48–50^. Given that negative emotional valence tends to elicit stronger right-lateralized responses^51–55^, increased right-prefrontal salience cooperation likely underlies a state of negativity bias, consistent with behavioral responses of sleep deprived individuals^56,57^. Enhanced limbic-salience coupling points to an amplification of interoceptive–affective signaling that would be expected to amplify salience attribution and also skew cognitive appraisal under sleep loss.

Oscillations in global efficiency demonstrate that functional brain networks are not static but dynamically reorganize over time^4^, occupying regionally distinct topological states shaped by interacting homeostatic and circadian influences. These findings indicate that circadian rhythmicity extends beyond neural activity to higher-order network topology, with both cortical and subcortical regions exhibiting unique temporal phases of reorganization. This raises the possibility of asynchronous phase alignment between regional networks, potentially underlying fluctuations in inter-network communication. Fluctuations within the right prefrontal and left amygdala networks align with the *Mind After Midnight* hypothesis, which posits that nocturnal wakefulness encapsulates homeostatic and circadian processes that bias cognition, emotion, and behavior toward dysregulation and increased risk-taking^2^. Network interactions between prefrontal and salience nodes are consistent with a shift in affective network dominance, reflecting altered emotional processing that significantly predicts subjective emotional and fatigue components. Finally, the use of optimized curve-fitting algorithms provides a data-driven framework for capturing nonlinear trajectories in functional outcomes. This approach offers a flexible model of how internal timekeeping and homeostatic sleep pressure jointly modulate large-scale functional organization and behavior across extended wakefulness.

## Methods

### Participants and procedures

Twenty participants (age 18–36) were recruited from Tucson, Arizona, using flyers and social media advertisements, and advertisements on university sponsored websites. Participant demographics are as follows: age ($\bar{x}=23.55$, σ = 4.65), education ($\bar{x}=15.06$, σ = 2.34), sex (*n*_male_ = 11, *n*_female_ = 9). Interested participants completed online screenings to exclude for psychiatric and other major health disorders, self-reported sleep disturbances, and to confirm MRI eligibility. Research protocols were conducted in accordance with ethical guidelines and approved by the University of Arizona Institutional Review Board. All ethical regulations relevant to human research participants were followed. Participants gave informed consent prior to participation in research activities.

Following the at-home screening, potential participants were brought into the lab for a preliminary assessment of neurocognitive status, drug testing, and a physical exam. Participants were then given a smartphone device for at-home monitoring of sleep-wake patterns via sleep diaries, and visual analogue scale of fatigue (VASF)^58^. Additionally, the Psychomotor Vigilance Task (PVT)^59^ was administered to participants twice a day (at 9 AM and 8 PM) to determine baseline reaction times. Participants with irregular sleep schedules or reaction times were excluded from the study. Eligibility for continuation in the study required 80% of all queries be completed.

The in-lab sleep-deprivation protocol was conducted at the University of Arizona Center for Sleep, Circadian, and Neuroscience Research. Participants arrived at 2:00 pm for drug testing and vital sign assessments. At 6:00 pm and 9:00 pm, they completed a battery of assessments which included the Karolinska Sleepiness Scale (KSS)^60^, VASF, symptoms inventory, and Visual Analog Mood Scale (VAMS)^61^. This combination of tests was administered throughout the remainder of the study and are subsequently referred to as the “standard battery 1”. After completing these tasks, participants followed their usual nighttime routine and went to bed at their scheduled bedtime of 10:30 PM.

**Fig. 5:**
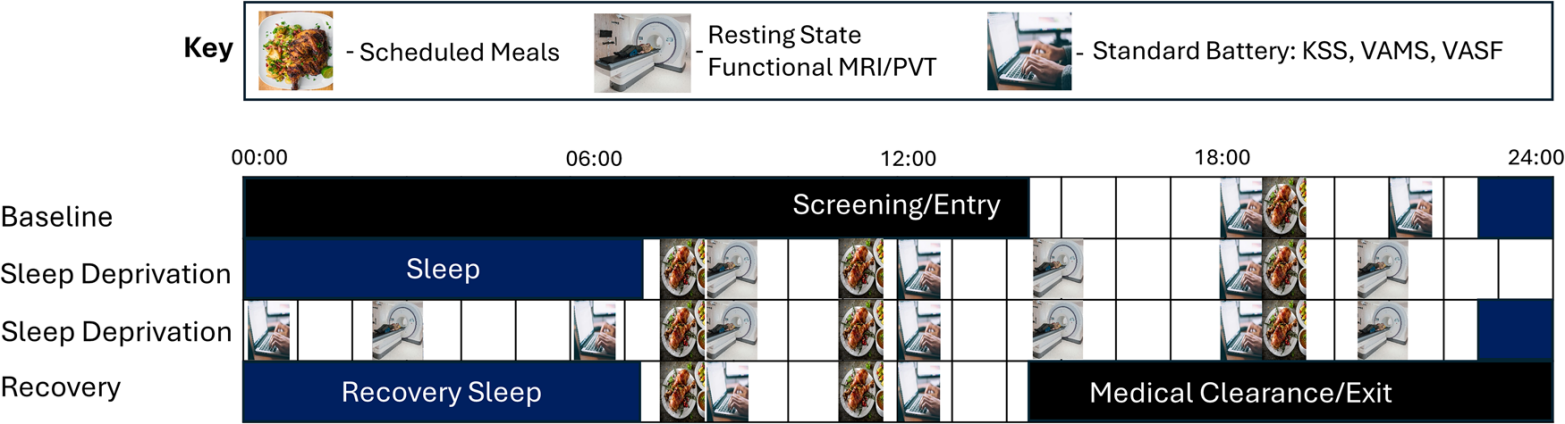
Schematic of the study protocol. Scheduled meals, functional MRI scans, and standard test batteries are represented graphically. Both standard batteries and resting-state MRI scans were conducted at 6-hour intervals. Twenty-four-hour clock times are shown at the top of the figure. Prior screening and post-study medical examination are illustrated by solid black bars. Blue bars represent baseline and recovery sleep episodes. Images were downloaded from (https://unsplash.com) and used under the Unsplash License. Photo credits: MRI [Accuray]; meal [Lilas Yohane]; computer, [Glen Carstens-Peters].

The 39-hour total sleep deprivation protocol began at 7:30 AM following the baseline sleep episode. MRI scans were conducted every six hours (seven in total) during this protocol, starting with a baseline scan at 8:30 AM. The standard battery 1 was also administered at six-hour intervals, beginning at approximately 12:30 pm. Mealtimes were provided at fixed times (8:00am, 11:00am, 6:30am). During free-time, participants were allowed to conduct their activity of choice, provided they did not lie down or rest, or perform physical activity. Recovery sleep began at 10:30 pm after 39-h of total sleep deprivation. After a nine‑hour recovery sleep, participants completed an additional round of cognitive assessment and underwent a medical examination by a licensed physician to confirm their health and safety before returning home. A schematic of the study protocol is shown in Fig. 5. Room illuminance was maintained at 100 lux for all participants.

### MRI acquisition

For each MRI session, participants were escorted to the University Translational Bioimaging Resource (Tucson, Arizona) where scans were acquired with a 3 T Skyra scanner (Siemens, Erlangen, Germany) equipped with a 32-channel head coil. Scanning sequences began with a localizer scout image, followed by a T1 weighted 3-D MPRAGE sequence (TR = 2.1 s, TE = 2.33 ms, flip angle = 12˚, n-slice = 176, Voxel = 1 mm^3^). Phase encoding direction was anterior to posterior (A»P). The images were acquired with a field of view (FoV) of 256 mm.

Functional images were acquired using a T2*- weighted echo-planar imaging (EPI) sequence. The imaging parameters were as follows: TR = 2000 ms, TE = 36.0 ms, flip angle = 90°, slice thickness = 2.0 mm, and voxel size = 2.0 mm³. A total of 60 slices were acquired in an interleaved order, with a field of view (FoV) of 240 mm. The phase encoding direction was A » P. The scan used GRAPPA parallel imaging with an acceleration factor of 2. The functional images were collected over 180 volumes with a total scan duration of approximately 6 min.

### MRI processing and analysis

Preprocessing was performed in SPM12^62^ alongside with the functional connectivity toolbox CONN (RRID:SCR_009550 Version: 22.v2407)^63,64^ using the default preprocessing pipeline^65^. Preprocessing included realignment to the first scan in the timeseries with correction of susceptibility distortion interactions using the SPM realign & unwarp procedure^66^. This procedure uses a least squares approach for a 6 parameter (rigid body) transformation^67^ and b-spline interpolation for distortion correction. Slice timing correction followed^68,69^, using sinc temporal interpolation to resample each slice BOLD timeseries to a common mid-acquisition time. Scan timing parameters were extracted from the metadata file for slice acquisition order determination. Outlier scans were identified using ART^70^ to pinpoint acquisitions with framewise displacement above 0.9 mm or global BOLD signal changes above 5 standard deviations^71,72^, and a reference BOLD image was computed for each subject by averaging all scans excluding outliers. Tissue segmentation was applied for gray matter, white matter, and CSF (cerebrospinal fluid) tissue classes, using the SPM12 default tissue probability map^73^. Both anatomical and functional scans were normalized to M.N.I. space with a 2 mm^3^ resolution^74,75^. Last, functional data were smoothed using spatial convolution with a Gaussian kernel of 8 mm full width half maximum (FWHM).

Following preprocessing, functional data were denoised using a standard denoising pipeline^65^ including the regression of potential confounding effects characterized by white matter timeseries, CSF timeseries, motion parameters and their first order derivatives^76^, outlier scans^72^, session and task effects and their first order derivatives, and linear trends within each session. A bandpass filter of the BOLD signal timeseries was applied between 0.008 and 0.09 Hz^77^. Noise components within white matter and CSF were estimated by computing the average BOLD signal as well as their principal components within each subject's eroded segmentation masks (CompCor)^78^, motion parameters, and outlier scans. From the number of noise terms included in this denoising strategy, the effective degrees of freedom of the BOLD signal after denoising were estimated across all subjects (average 34.2)^71^. Additional quality assurance analyses were conducted based on global signal changes and distribution of functional connectivity values to determine if there were any significant outliers in the data. One subject was omitted from analyses due to incomplete data (*n* = 19).

First and second level analyses are conducted using a general linear model (GLM) to calculate function connectivity^65^. First level analyses use subject-specific covariates for denoise components and motion artifact to reduce error variance not directly representative of underlying gray-matter neurovascular coupling. Functional connectivity is then calculated as the bivariate correlation between the BOLD signal for the region of interest and peripheral regions modeled as independent and dependent variables, respectively. Correlational coefficients were Fisher transformed to adhere to statistical assumptions of normality. Group-level measures of functional connectivity similarly use a GLM in which the design matrix encodes explanatory variables (e.g., condition, timepoint, and covariates) and the subject-level connectivity maps serve as the dependent variables. A coefficient matrix is estimated using an ordinary least squares approach to determine the strength and direction of the relationship between each explanatory variable in the design matrix and each functional connectivity value. Hypotheses were evaluated using multivariate parametric statistics with random-effects across subjects and sample covariance estimation across multiple measurements. Inferences were performed at the level of individual clusters (groups of contiguous voxels). Cluster-level inferences were based on parametric statistics from Gaussian Random Field theory^65,79^. Results were thresholded using a combination of a *p* < 0.001 voxel-level threshold, and a false discovery rate corrected p-FDR (false discovery rate) < 0.05 cluster-size threshold^80^.

Using this statistical framework, seed-based connectivity maps and ROI-to-ROI (region of interest) connectivity matrices were estimated characterizing the patterns of functional connectivity. The ROIs included a combinations of regions from the Harvard-Oxford atlas (RRID:SCR_001476) and the Automated Anatomical Labeling atlas^81^ with a total of 165 regions. A bilateral thalamic ROI was also added to confirm laterality of effect. Functional connectivity strength was represented by Fisher-transformed Pearson’s correlation coefficients from a weighted GLM, defined separately for each pair of seed and target areas, modeling the association between their blood oxygenation level dependent (BOLD) signal timeseries.

Graph theory analysis was then applied to functional connectivity data to enable higher order network properties such as global efficiency. Calculations for Global efficiency first require a connectivity matrix representing a network graph of positive connectivity between regions of interest. Cost (Eq. 1) is first calculated from a binary adjacency matrix A_i,j_ by applying a proportion-based connectivity threshold set at 0.15, thereby retaining the dominant connections within the regional network. Measures of global efficiency were then calculated between nodes (regions) and network paths (retained connections) from the individual adjacency matrices. Global efficiency (Eq. 2) is calculated as the average of the inverse shortest path lengths between the node of interest and all other connected nodes in the network^65^. Distance is represented by the shortest number of steps (edges) connecting two nodes in the regional network (D_i,j_). N represents the number of nodes in the network.

1 $${{Cost}}_{i}=\frac{{\sum }_{i}{A}_{i,j}}{N-1}$$

2 $${{Global}\,{Efficiency}}_{i}=\frac{{\sum }_{j\ne i}1/{D}_{i,j}}{N-1}$$

Global efficiency coefficients take a value between 0 and 1 which effectively represent how easily a brain region (node_i_) can communicate with any other region in the network (node_j_), either directly or through intermediate connections. A global efficiency value of 1 would indicate that the node is directly connected to all other nodes in the network via paths of equal and minimal length, representing an idealized condition of maximal integration.

While global efficiency conveys topological properties of network states, it does not quantify the dominant connectivity features that contribute to network reorganization. To further clarify specific changes within prefrontal and amygdala networks, changes in functional connectivity were quantified between timepoints of maximum and minimum network efficiency.

A subset of this data was previously analyzed and published focusing particularly on thalamic-cortical dysconnectivity^30^. Here, we applied an alternative approach that examines lateralized circadian dynamics and potential compensatory network effects, with particular emphasis on rhythms in the frontolimbic and sensorimotor networks. The analyses and figures presented here have not been published previously.

### Statistics and reproducibility

ROI-ROI connectivity matrices of global efficiency for each participant were analyzed in MATLAB 2023a^82^. Model fitting was performed using MATLAB’s *lsqcurvefit* algorithm in the optimization toolbox. This algorithm takes both the observed data (y data_i_) and a defined function F(x, x data_i_) with adjustable parameters (x). These parameters are then adjusted to minimize the sum of squared residuals (Eq. 3) between the model predictions and the observed data:

3 $${\min }_{x}{\sum }_{i}{F\left(x,x{{data}}_{i}\right)-y{{data}}_{i}\left)\right.}^{2}$$

In other words, parameter adjustments are guided by a local approximation of residual error, constrained within a bounded “trust region”^83^ based on the interior-reflective Newton method^84^. Optimal parameter adjustments are determined by finding a local minimum in the least-squares cost function relative to the observed data^85^. In effect, this approach finds the collection of function parameters that maximize function fit to the data without a priori assumptions of oscillatory dynamics.

For applications of circadian modeling of brain connectivity data, the objective function for optimization was defined using the circadian cosine model (Eq. 4)^86–88^:

4 $$Y=M+A \, cos \left(\frac{2\pi }{\tau }* (t-\phi )\right)$$

Four variables were defined as adjustable parameters which included the mesor (M), the rhythm-adjusted mean level of the signal; amplitude (A), the magnitude of oscillatory deviation from the mean; period (τ), the duration of one full oscillatory cycle; and acrophase (φ), the timing of the peak of the oscillation. Parameters M, A, and φ were left unbounded to maximize best-fit optimization. The period parameter was constrained within reasonable bounds to enable detection of spatial transformations reflecting homeostatic and circadian interactions, while accounting for aliasing effects (24–39 h ± σ_τ_). Parameters were fit to group-means of global efficiency at each timepoint. To evaluate model fit, we compared the variance explained by the fitted nonlinear model to that of a null model. An F-statistic was computed comparing the variance explained by the fitted model to the residual variance, adjusted for the number of model parameters and sample size. This approach is mathematically equivalent to the F-test described in cosinor analyses, where the model sum of squares is compared to the residual sum of squares using appropriate degrees of freedom^89^.

Regions for modeling were selected in a hypothesis-driven manner. Sensorimotor and limbic networks are involved in cognitive processes that show both circadian and wake-dependent deficits. To determine whether topological measures are effective indicators of behavioral indices, confirmatory mixed linear models were conducted on areas with significant oscillations in global efficiency. Random effects across participants were defined to account for subject-specific baseline differences, allowing a more direct estimation of the relationship between network efficiency and behavioral variables while appropriately handling repeated measures. Mixed linear effect models were calculated in R studio^90^.

### Behavioral measures

Psychomotor vigilance tests were administered on a computer using PsychoPy software (version 2024.2.1)^91^. Mean reaction time data were calculated using predefined thresholds between 150 and 4999 ms. Responses below 150 ms were classified as false starts. Analogue scales of fatigue and emotion were collected and scored by trained technicians. Fatigue components were scored in accordance with previous research^58^.

As sleep deprivation can induce complex mood states, negative emotional components were isolated and summed from the VAMS. Inter-individual differences in baseline emotional state and emotional evaluative capacity were further addressed via within-subject normalization. Other sub-components such as energetic, sad, and tiredness, were not included given their multicollinearity with subjective fatigue^92,93^ which was modeled independently using the VASF data. Changes in each component are assessed in 12-h intervals using within subject T-tests to target opposing circadian phase. Effect sizes (Cohen’s d) are calculated as the standardized mean difference divided by the pooled standard deviation of the two timepoints.

### Limitations

Sampling resolution is a critical consideration in chronobiological studies for detecting rhythmicity with sufficient precision. Due to scanner availability and participant burden, functional imaging sessions were acquired at six-hour intervals. While this sampling frequency is adequate to capture infradian fluctuations (i.e., periodicities exceeding 24 h), it limits the resolution needed to reliably detect shorter cycles. Thus, detection of shorter periods should be interpreted with caution, as there is a possibility of aliasing.

Individual variability with respect to differences in functional network localization, or resilience to sleep deprivation likely contributes to sample variance. Thus, it is unclear whether apparent laterality of some effects represent inherent meaningful heterogeneity in oscillatory dynamics or individual differences of functional network localization. Future research analyzing circadian trends in functional networks would benefit from considering individualized profiles.

### Reporting summary

Further information on research design is available in the Nature Portfolio Reporting Summary linked to this article.

## Supplementary information

Transparent Peer Review file

Supplementary Information

Description of Additional Supplementary files

Supplementary Data 1

Reporting Summary

## Data Availability

Data may be made available upon reasonable request to the corresponding author, provided the data being shared adheres to participant privacy rights. Source data for graphs and figures have been submitted to the University of Arizona’s Research Data Repository (ReDATA)^94^ under 10.25422/azu.data.32069517.

## Code availability

Documentation for code and methods of the lsqcurvefit function can be found at https://www.mathworks.com/help/optim/ug/lsqcurvefit.html as part of the optimization toolbox. Function specification and parameter definitions are clearly described in the methods section.
