# Supplementary material for: Oscillatory network efficiency predicts mood and fatigue during sleep deprivation
Source: Commun Biol. 2026 May 16;9:1041. doi: 10.1038/s42003-026-10250-8 (PMC13438542; doi:10.1038/s42003-026-10250-8)
Supplement: Supplementary file 1 [file 42003_2026_10250_MOESM1_ESM.pdf]

nature portfolio

Peer Review File

Oscillatory Network Efficiency Predicts Mood and Fatigue During Sleep Deprivation

Corresponding Author: Dr David Negelspach

This file contains all reviewer reports in order by version, followed by all author rebuttals in order by version.

Version 0:

Reviewer comments:

Reviewer #1

(Remarks to the Author)
Dear colleagues!
The present article is devoted to the study examines changes in the functional connectivity of brain networks during sleep deprivation. It analyzes how circadian rhythms and homeostatic sleep pressure influence the topology of frontal, limbic, and sensorimotor networks using fMRI data from 20 participants during a 39-hour sleep deprivation protocol. The study shows that the brain's functional structure is not static but dynamically restructured under the combined influence of circadian rhythms and homeostatic sleep pressure (sleep demand). These changes in brain network topology predict fluctuations in mood, fatigue, and attention in sleep-deprived individuals. Another interesting point is the fact that different areas of the brain have unique temporal phases of their reorganization, which creates asynchrony in their work and explains fluctuations in the performance of various cognitive and emotional functions.
The paper can be accepted for publication with the following additions:
• How are the graphs on the left and right in Figure 3 related?
• How do the connections between different brain regions change? Is there a data table or something like that?
• How was this Global efficiency coefficient calculated? A slightly more detailed explanation from a mathematical point of view is needed.
• It seems to me that with different topologies (in different brain states) one could try to create a set of maps-diagrams of interactions between areas, to more clearly demonstrate the connectivity.
When reading this article, one gets the general impression that the description of the analysis, methods, and graphs is insufficient. However, this does not affect the authors' main idea or result.
While these changes do not affect the work's scientific merit, they will significantly improve its perception and compliance with the journal's publication requirements.

Best wishes, G. A. Guyo

Reviewer #2

(Remarks to the Author)
This manuscript investigates the relationship between brain network topology and circadian-related temporal dynamics. The authors conducted a ~36–39-hour total sleep deprivation experiment in 20 participants, acquiring seven resting-state fMRI scans and six repeated behavioral assessments (“standard battery 1”). They applied a nonlinear least-squares curve-fitting approach to model rhythmic fluctuations in behavioral outcomes and network efficiency measures. The authors report oscillatory patterns in network efficiency and ROI-to-ROI connectivity, with prominent effects in four regions (right frontal pole, left thalamus, left amygdala, and right postcentral gyrus). They further report significant associations between behavioral measures (PVT, VAS-F, VAMS) and efficiency in the postcentral gyrus, amygdala, and frontal pole. Overall, the study suggests that time-varying network efficiency may provide a systems-level neural framework for understanding vulnerability to affective and performance degradation during sleep loss.

While the topic is interesting, several concerns should be addressed:

1.A schematic timeline or flow chart would greatly improve readability and help readers understand the study design

currently described around lines 121–127.
2.The manuscript mentions both the Harvard–Oxford atlas and the Automated Anatomical Labeling (AAL) atlas. Please clarify whether these atlases were used independently or combined (e.g., subcortical ROIs from Harvard–Oxford and cortical ROIs from AAL). In addition, please report the final number of ROIs included in the analysis.
3.Please specify the connectivity thresholding strategy and matrix type used to compute global/nodal efficiency (e.g., binary vs weighted; proportional thresholding/sparsity; treatment of negative correlations). More methodological detail is needed for reproducibility.
4.It appears that the right frontal pole and left amygdala were selected as seed regions based on the efficiency results (not quite sure), and their connectivity with other atlas regions was compared between time points corresponding to local maxima and minima of regional efficiency. Please clarify this workflow explicitly in the Methods, including the statistical model and the multiple-comparisons correction procedure.
5.In the “Subject-level Mixed Linear Modeling” section, only selected ROIs are related to behavioral outcomes (PVT, fatigue, VAMS). Please provide a clear rationale for selecting these regions and clarify whether this selection was hypothesis-driven or data-driven.
6.The manuscript would benefit from a dedicated Methods subsection describing preprocessing and statistical analyses for behavioral measures (e.g., PVT reaction time metrics, VAS-F scoring, VAMS scoring), including how repeated measures were modeled.
7.In line 374: “This finding extends prior research on thalamocortical involvement of arousal and sensorimotor processing”. The link between thalamocortical connectivity and sensorimotor processing is not clearly supported in the surrounding text. Please either provide appropriate evidence/citations and expand the discussion, or revise the statement to better match the presented results.
8.Please include confidence intervals (or standard error bands) for the regression lines in the scatter plots (Figure 4).
9.What is the difference between “circadian rhythoms” and “infradian rythms” mentioned in abstract (line 8)?.
10.The abbreviations and symbols in the global efficiency formula (around line 199) should be defined in the accompanying paragraph. Additionally, please clarify the meaning of the other formula shown around line 198 and how it relates to the efficiency metric used in the analyses.
11.Ensure consistent terminology and abbreviations throughout. For example, the visual analogue scale of fatigue is denoted as “VASF” in some places (e.g., lines 108 and 251) and “VAS-F” elsewhere. Please standardize these terms throughout the manuscript.

Reviewer #3

(Remarks to the Author)
Summary
The study investigates the neural predictors of mood and fatigue during sleep deprivation. They propose that a metric called oscillatory network efficiency is a marker of these neural predictors. They claim that with the algorithm and overall methodology, they can distinguish between the circadian and homeostatic processes. They find regionally specific patterns of sleep deprivation vulnerability.
General comments
The equations of the graph theory analyses are not denoted properly: Eq 1, Eq 2 and are not referenced in the text. Furthermore, the equations are explained, but there is no link between the variable name and what they denote, and some variables are not explained as to what they refer to. This should be corrected for clarity and to avoid wrong assumptions. The second equation for the optimization is also not marked and referenced properly, although the elements in this case are explained.
Can the authors add a plot of the non-normalized VAMS in the supplementary materials? It would be good to see the raw variability in mood in the sample, as this is something that is also commented on in the discussion.
Is there a reference where the method for the functional connectivity calculator is introduced?
The way the GLM is used lacks clarity, such as the full meaning of “other subject-level identifiers as independent variables”. The authors should more clearly explain the first-level analysis part and second level group analyses. Furthermore, they should state the advantage of using this method compared to the standard Pearson correlation.
Major methodological comments
The authors use a large spatial smoothing of 8mm. In my opinion, this should be avoided because they are looking at smaller regions like the amygdala, and the smoothing will just deepen an already existing problem of partial volumes. The authors should either give a good explanation of why this is not a problem or redo the preprocessing without the smoothing.
Minor comments
The authors should add the modality used in the abstract, in this case, fMRI.
For clarity, the terms mesor and acrophase should be explained.
Figures are discussed in the text but are not referenced (eg. Fig 1a etc).
Is there a reason why the table is put in the figure? This data should be in the main text or in a separate table.
For clarity, the abbreviations used in figures can be put in the figure descriptions, but I leave this to be finally decided based on the journal guidelines.
Conclusion
The experimental study is complex, and I congratulate the authors on their work! The introduction and discussion are written clearly, with a few improvement points on the results and materials.
The major methodological flaw that I can see is the smoothing, and I hope it can be properly addressed by the authors.

I want to state that no AI was used in any stage of this review.

Version 1:

Reviewer comments:

Reviewer #1

(Remarks to the Author)
This updated version of the article represents a significant advancement compared to the previous edition. The authors have taken into account virtually all of the reviewers' comments, significantly improved methodological transparency, expanded the description of statistical approaches, and improved the visualization of results. This work makes an important contribution to our understanding of the dynamics of functional brain networks during sleep deprivation, as well as their relationship with cognitive and affective measures.
The authors have detailed the procedures for data preprocessing, calculating global efficiency, selecting ROIs, thresholding connectivity matrices, and statistical analysis. Additional explanations have been provided for formulas, notations, and terminology. A study protocol diagram has been added (Figure 1), figure captions have been improved, confidence intervals have been added to the graphs, and a separate figure has been added to illustrate connectivity changes (Supplementary Materials). The methods for multiple comparisons, mixed linear models, and criteria for selecting regions for analysis have been refined. This improves the reproducibility and interpretability of the results.
I would like to note that all the reviewers' key questions (regarding terminology, formulas, atlas selection, and behavioral data processing) were comprehensively answered and reflected in the text. I recommend this article for publication.

Reviewer #2

(Remarks to the Author)
After reviewing the rebuttal and revised manuscript, I have one remaining minor comment: in Figure 5, the y-axis label is still shown as “VAS-F” and should be corrected to “VASF.” Otherwise, I have no further comments.

Reviewer #3

(Remarks to the Author)
The authors diligently addressed the comments and improved the quality of the manuscript.

Open Access This Peer Review File is licensed under a Creative Commons Attribution 4.0 International License, which permits use, sharing, adaptation, distribution and reproduction in any medium or format, as long as you give appropriate credit to the original author(s) and the source, provide a link to the Creative Commons license, and indicate if changes were made.
In cases where reviewers are anonymous, credit should be given to 'Anonymous Referee' and the source.
The images or other third party material in this Peer Review File are included in the article’s Creative Commons license, unless indicated otherwise in a credit line to the material. If material is not included in the article’s Creative Commons license and your intended use is not permitted by statutory regulation or exceeds the permitted use, you will need to obtain permission directly from the copyright holder.

To view a copy of this license, visit https://creativecommons.org/licenses/by/4.0/

Response to Reviewer Comments

We sincerely thank the reviewers for their thoughtful and constructive critiques. Their insights have significantly strengthened the manuscript by improving scientific rigor and methodological clarity. Each comment has been carefully considered and addressed below. The revised manuscript reflects these improvements and clarifies the methodology, figures, and analyses throughout.

Response to Reviewer 1 Comments

Comment 1: *How are the graphs on the left and right in Figure 3 related?*
Response 1: In the initial figure, the line graph (left) showed the timepoint comparison on the global efficiency curve. To improve clarity for this figure (now figure 4), the line plot has been removed and the comparison is stated directly in the Results section (page 13, lines 359–364 & 376-377).

Comment 2: *How do the connections between different brain regions change? Is there a data table or something like that?*
Response 2: We have provided the raw data for connectivity associated with the transition from rested to sleep deprived in the well-rested condition. Please see the "Supplementary Data Raw Connectivity Changes" file.

Comment 3: *How was this Global efficiency coefficient calculated? A slightly more detailed explanation from a mathematical point of view is needed*
Response 3: We concur that additional detail is warranted regarding the mathematics of global efficiency and have expanded the Methods section to further explain how global efficiency is calculated (page 7, lines 220-232).

Comment 4: *It seems to me that with different topologies (in different brain states) one could try to create a set of maps-diagrams of interactions between areas, to more clearly demonstrate the connectivity.*
Response 4: Showing a set of diagrams or figures that clearly show changes in topological state at each timepoint would be helpful to demonstrate temporal trends in connectivity. Unfortunately, visualizing changes in the group topological state would not be possible, as each participant contains a unique binary adjacency matrix, which cannot be combined into an average representation. The changes in connectivity, however, are the primary contributing sources to individual changes in topological state and can be provided. To address this, a supplementary figure has been added that summarizes regional network connectivity. Please see the "Supplementary Figure Frontolimbic Network States" file.

Comment 5: *When reading this article, one gets the general impression that the description of the analysis, methods, and graphs is insufficient. However, this does not affect the authors' main idea or result. While these changes do not affect the work's*

scientific merit, they will significantly improve its perception and compliance with the journal's publication requirements
Response 5: We fully agree with the reviewer's constructive remarks. In addition to the modifications described above, the Methods sections detailing the statistical frameworks, optimization algorithm, and global efficiency calculations have been expanded and revised to improve clarity and reproducibility. Please see the highlighted sections on pages 6–9, as well as the modifications made in response to related reviewer comments below.

Response to Review 2 Comments

Comment 1: *A schematic timeline or flow chart would greatly improve readability and help readers understand the study design currently described around lines 121–127.*
Response 1: We agree that a schematic would improve the interpretation of study activities. A new Figure 1 has been added to show the schematic overview of the study design (pages 4-5, lines 133–139).

Comment 2: *The manuscript mentions both the Harvard–Oxford atlas and the Automated Anatomical Labeling (AAL) atlas. Please clarify whether these atlases were used independently or combined (e.g., subcortical ROIs from Harvard–Oxford and cortical ROIs from AAL). In addition, please report the final number of ROIs included in the analysis.*
Response 2: The combination of the Harvard–Oxford atlas and the Automated Anatomical Labeling atlas (AAL) has been made clearer in the manuscript along with the total number of ROIs (page 7, lines 212–214).

Comment 3: *Please specify the connectivity thresholding strategy and matrix type used to compute global/nodal efficiency (e.g., binary vs weighted; proportional thresholding/sparsity; treatment of negative correlations). More methodological detail is needed for reproducibility.*
Response 3: The methods section describing the global efficiency calculations has been revised to provide additional methodological detail, including a clearer description of the proportion-based thresholding steps (page 7, lines 220–230).

Comment 4: *It appears that the right frontal pole and left amygdala were selected as seed regions based on the efficiency results (not quite sure), and their connectivity with other atlas regions was compared between time points corresponding to local maxima and minima of regional efficiency. Please clarify this workflow explicitly in the Methods, including the statistical model and the multiple-comparisons correction procedure.*
Response 4: The reviewer is correct that the targeted connectivity analyses were conducted to expand upon the modeling approach. These analyses were performed with the goal of clarifying which specific network connections were driving the dominant

graph-theory changes. Because global efficiency reflects only topological network properties, our primary objective was to quantify the connectivity changes underlying these shifts in network state, as reflected in the revised Methods section (pages 8, lines 238–243). Information regarding multiple-comparison correction and the statistical framework has been added to the figure legend (page 13, lines 383–384).

Comment 5: *In the "Subject-level Mixed Linear Modeling" section, only selected ROIs are related to behavioral outcomes (PVT, fatigue, VAMS). Please provide a clear rationale for selecting these regions and clarify whether this selection was hypothesis-driven or data-driven.*
Response 5: We agree that a direct explanation of the region selection criteria should be included. The methods section has been updated to reflect this change and directly states the hypothesis-driven manner of predefined regional network analysis and confirmatory mixed-effects modeling (page 9, lines 280–284).

Comment 6: *The manuscript would benefit from a dedicated Methods subsection describing preprocessing and statistical analyses for behavioral measures (e.g., PVT reaction time metrics, VAS-F scoring, VAMS scoring), including how repeated measures were modeled.*
Response 6: A separate section describing the limited preprocessing and scoring of the data along with the analysis for timepoint comparison has been added. Regarding repeated measures, mixed-effects linear models with random intercepts across subjects were used, which is a standard approach for analyzing repeated within-subject data and is described in the previous section (page 9, lines 286–298).

Comment 7: *In line 374: "This finding extends prior research on thalamocortical involvement of arousal and sensorimotor processing". The link between thalamocortical connectivity and sensorimotor processing is not clearly supported in the surrounding text. Please either provide appropriate evidence/citations and expand the discussion or revise the statement to better match the presented results.*
Response 7: The description of thalamic involvement in cortical arousal modulation and sensorimotor processing has been expanded with a particular emphasis on thalamic modulation characterized by sustained attention and psychomotor vigilance responses from the cited research (page 15, lines 442–451).

Comment 8: *Please include confidence intervals (or standard error bands) for the regression lines in the scatter plots (Figure 4).*
Response 8: Confidence intervals have been added to the regression model graphs (page 14, lines 404-405).

Comment 9: *What is the difference between "circadian rhythms" and "infradian rhythms" mentioned in abstract (line 8)?*

Response 9: Infradian rhythms are oscillations that take longer than 24 hours to complete, such as over the course of days. A brief definition reflecting this has been added to the abstract (page 1, line 8).

Comment 10: *The abbreviations and symbols in the global efficiency formula (around line 199) should be defined in the accompanying paragraph. Additionally, please clarify the meaning of the other formula shown around line 198 and how it relates to the efficiency metric used in the analyses.*
Response 10: Variables in the global efficiency and cost equations have been referenced and defined in the methods section. A direct connection between the cost threshold and global efficiency has been included in the description of calculations (page 7, lines 223–230).

Comment 11: *Ensure consistent terminology and abbreviations throughout. For example, the visual analogue scale of fatigue is denoted as "VASF" in some places (e.g., lines 108 and 251) and "VAS-F" elsewhere. Please standardize these terms throughout the manuscript.*
Response 11: To ensure consistency with abbreviations in the manuscript, VAS-F has been changed to VASF in all instances.

Response to Review 3 Comments

Comment 1: *The equations of the graph theory analyses are not denoted properly: Eq 1, Eq 2 and are not referenced in the text. Furthermore, the equations are explained, but there is no link between the variable name and what they denote, and some variables are not explained as to what they refer to. This should be corrected for clarity and to avoid wrong assumptions. The second equation for the optimization is also not marked and referenced properly, although the elements in this case are explained.*
Response 1: Each equation has been labeled and properly referenced in the methods section. Specific variables have been further explained in the context of their contribution to global efficiency calculations derived from functional connectivity. Corresponding references have been made in text in the methods section. Each variable term has also been included in the methods description for calculations (pages 7–8, lines: 222, 226, 231, 232, 252–256, 264, 265).

Comment 2: *Can the authors add a plot of the non-normalized VAMS in the supplementary materials? It would be good to see the raw variability in mood in the sample, as this is something that is also commented on in the discussion.*
Response 2: We share the opinion that conveying variability in behavioral measures is extremely important. To address this point, we have plotted variability in the behavioral

measures, including VAMS, vis-à-vis box plots at each timepoint (page 10, lines 318–319). Including raw VAMS scores would not aid interpretation because between-subject differences in baseline ratings and emotional regulation are unrelated to the effects of interest; normalization removes this between-subject variability and facilitates comparison of within-subject changes across sleep deprivation. Normalized VAMS scores are included in the “Supplementary Data Behavioral Measures PVT VASF VAMS” file.

Comment 3: *Is there a reference where the method for the functional connectivity calculator is introduced?*
Response 3: The citations for referencing the functional connectivity toolbox has been confirmed in the main text (references 28 & 29).

Comment 4: *The way the GLM is used lacks clarity, such as the full meaning of “other subject-level identifiers as independent variables”. The authors should more clearly explain the first-level analysis part and second level group analyses. Furthermore, they should state the advantage of using this method compared to the standard Pearson correlation.*
Response 4: We have revised the Methods section to clarify both the functional connectivity estimation procedure and the hierarchical GLM framework used for statistical inference. The correlation used is Pearson correlation which is then fisher transformed to more appropriately abide assumptions of normality. This has been specified in the methods section (page 6, lines 190–202).

Comment 5: *The authors use a large spatial smoothing of 8mm. In my opinion, this should be avoided because they are looking at smaller regions like the amygdala, and the smoothing will just deepen an already existing problem of partial volumes. The authors should either give a good explanation of why this is not a problem or redo the preprocessing without the smoothing.*
Response 5: The reviewer suggests a smaller smoothing kernel would be preferred for functional connectivity analysis. This aspect was carefully considered prior to the analysis. Smoothing is beneficial for the analysis of functional data, as transient signal can be confounded by significant outliers due to magnetic inhomogeneity, artifact, or transient signal spikes. Gaussian smoothing allows data to be weighted according to regional datasets, thereby improving the signal-to-noise ratio (SNR). Smaller smoothing kernels provide increased granularity but do so at the risk of regional inhomogeneity of results and reduced SNR. Less smoothing is typically preferred in situations such as seed-to-voxel connectivity, as this provides higher spatial accuracy. In this case, a larger smoothing kernel was chosen to be more compatible with ROI-ROI connectivity, which considers regionally averaged time series; a larger kernel therefore reduces between-subject variability. In addition, larger smoothing kernels reduce inter-subject anatomical variability, which can further introduce noise when comparing connectivity across

subjects. Independent research examining the impact of spatial smoothing on group-level functional connectivity indicates that graph-theoretical metrics remain relatively stable across moderate smoothing kernel sizes (Candemir C. (2023). Spatial Smoothing Effect on Group-Level Functional Connectivity during Resting and Task-Based fMRI. *Sensors (Basel, Switzerland)*, *23*(13), 5866.)

Comment 6: *The authors should add the modality used in the abstract, in this case, fMRI.*
Response 6: Modality (fMRI) was included in the abstract (page 1, line 9).

Comment 7: *For clarity, the terms mesor and acrophase should be explained.*
Response 7: Parameters included in the optimization formula have been individually defined (page 8–9, lines: 267–269)

Comment 8: *Figures are discussed in the text but are not referenced (eg. Fig 1a etc).*
Response 8: Figure panels have been referenced in text (page 10, lines 307–308; page 14, lines 394, 398, & 402).

Comment 9: *Is there a reason why the table is put in the figure? This data should be in the main text or in a separate table.*
Response 9: Table 1 has been changed to a separate table in the main text document.

Comment 10: *For clarity, the abbreviations used in figures can be put in the figure descriptions, but I leave this to be finally decided based on the journal guidelines.*
Response 10: Abbreviations of behavioral variables have been expanded in full for clarity. Explanations for the L. and R. abbreviations were added to Figure 3 (page 12, line 350).
