# Supplementary material for: Oscillatory network efficiency predicts mood and fatigue during sleep deprivation
Source: Commun Biol. 2026 May 16;9:1041. doi: 10.1038/s42003-026-10250-8 (PMC13438542; doi:10.1038/s42003-026-10250-8)
Supplement: Supplementary file 2 [file 42003_2026_10250_MOESM2_ESM.pdf]

Supplementary Figure 1

Supplementary Figure 1 | Panels display regional functional connectivity patterns for the right frontal pole (a-g) and left amygdala (h-n) at each timepoint throughout sleep deprivation. Significant ROI-ROI connections are shown using ring diagrams. Lines (edges) indicate significant functional connections between regions and are color-coded by the direction and magnitude of the statistical effect. Heatmaps for corresponding T-statistics of each connection are displayed (top right). Panel o provides the key for abbreviated anatomical region labels used in the ring diagrams.

a.) Right Frontal Pole Functional Connectivity (1 h awake, 8:30 am)

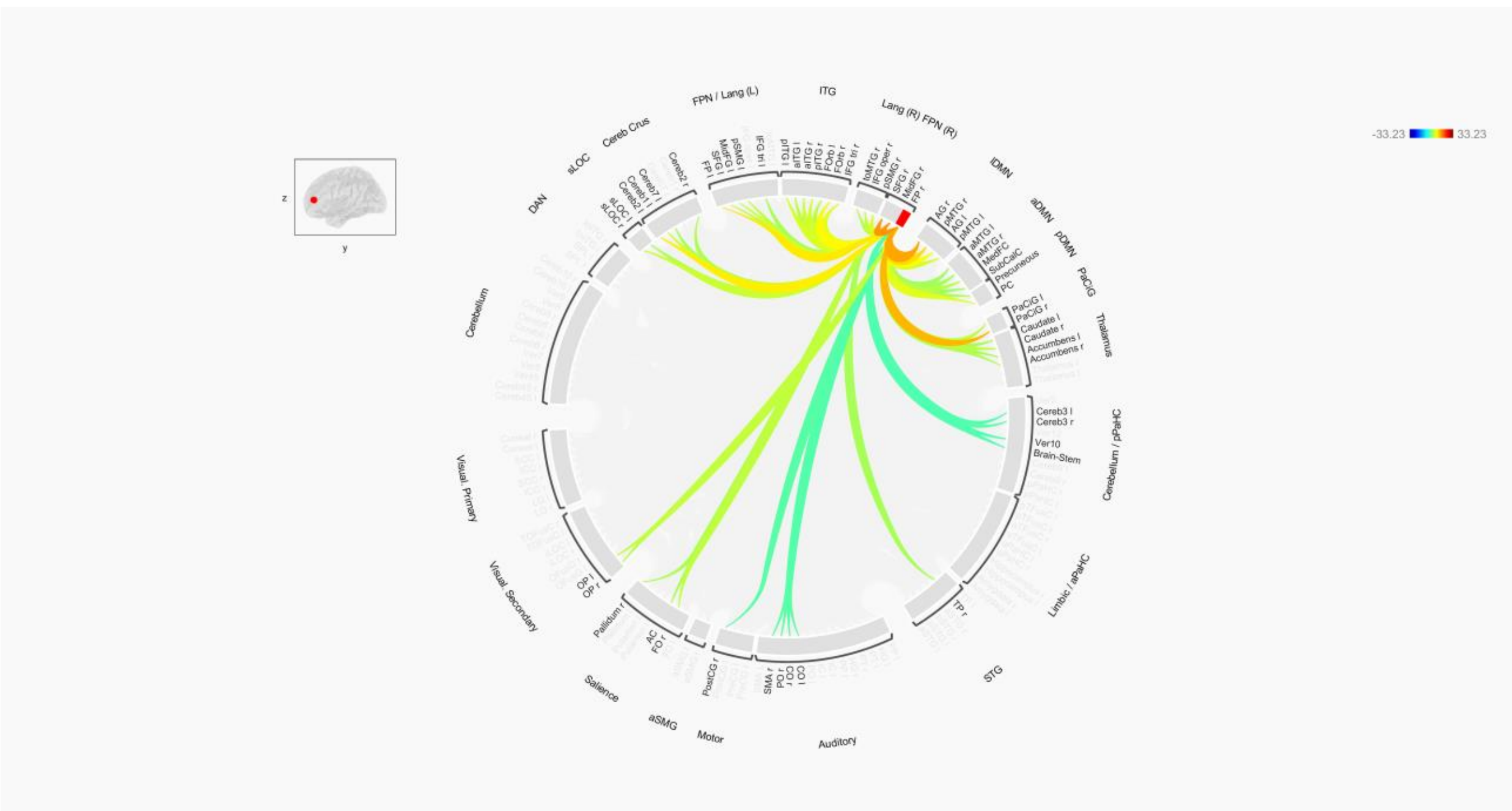

b.) Right Frontal Pole Functional Connectivity (7 h awake, 2:30 pm)

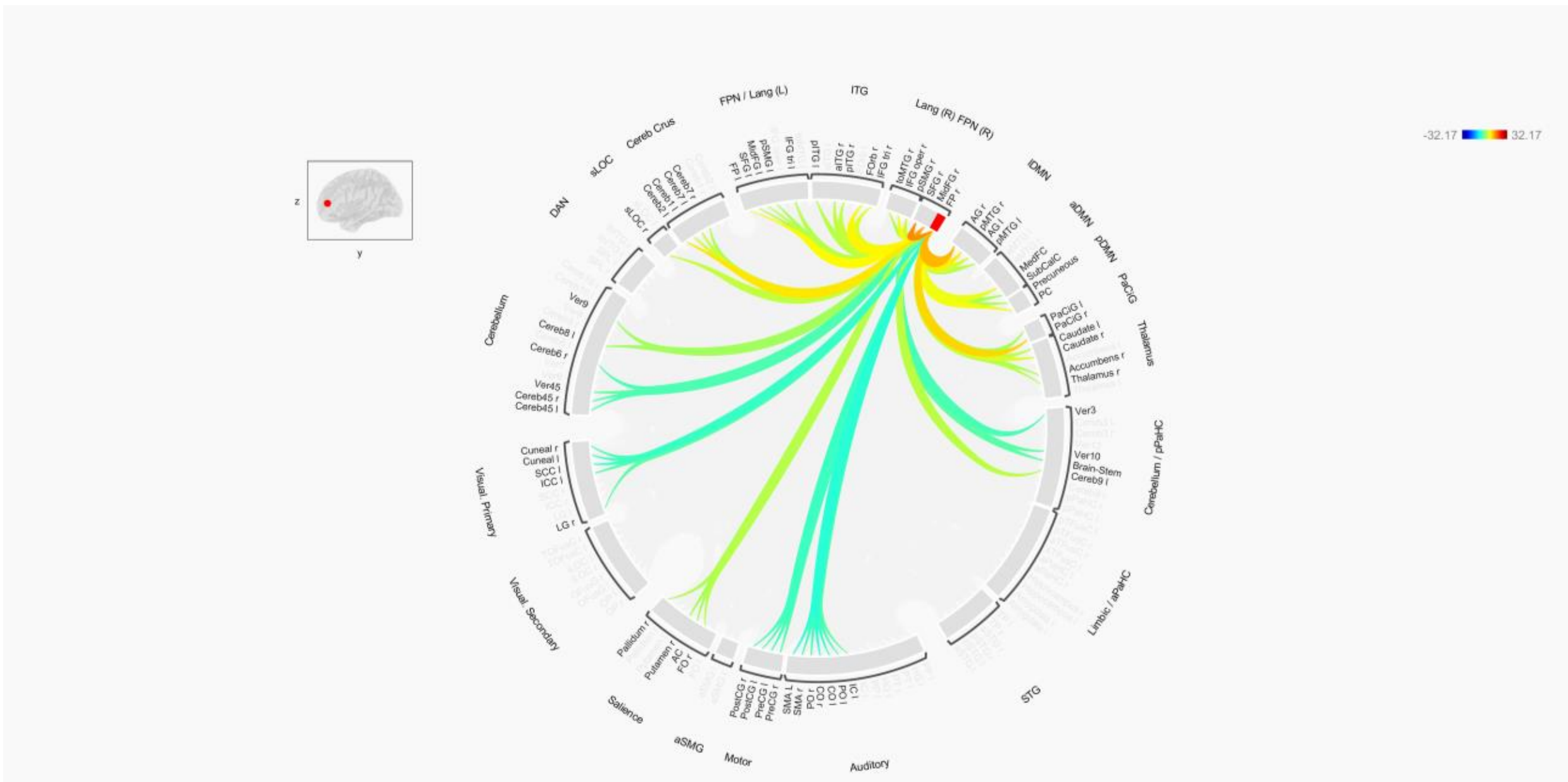

c.) Right Frontal Pole Functional Connectivity (13 h awake, 8:30 pm)

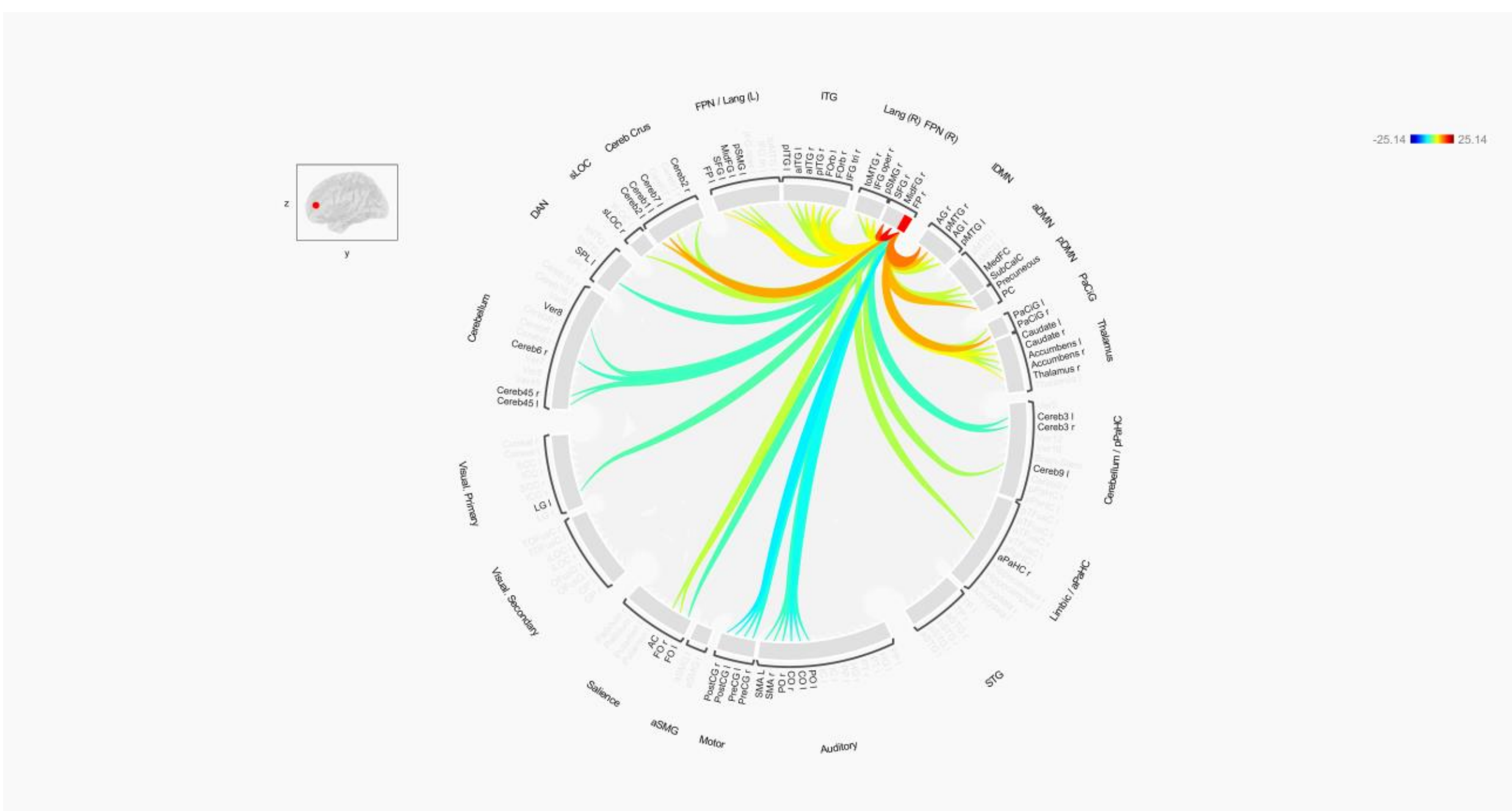

d.) Right Frontal Pole Functional Connectivity (19 h awake, 2:30 am)

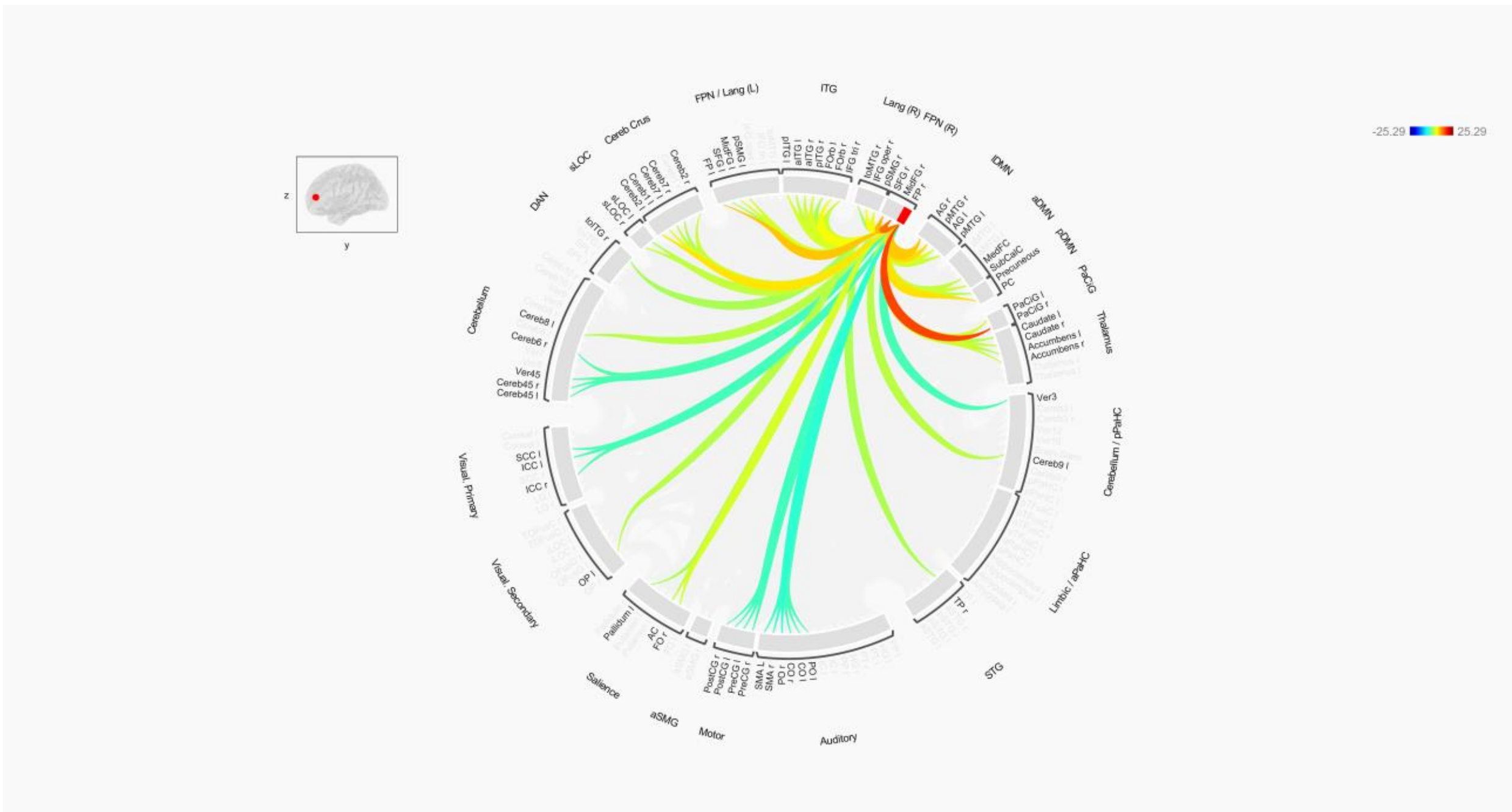

e.) Right Frontal Pole Functional Connectivity (25 h awake, 8:30 am)

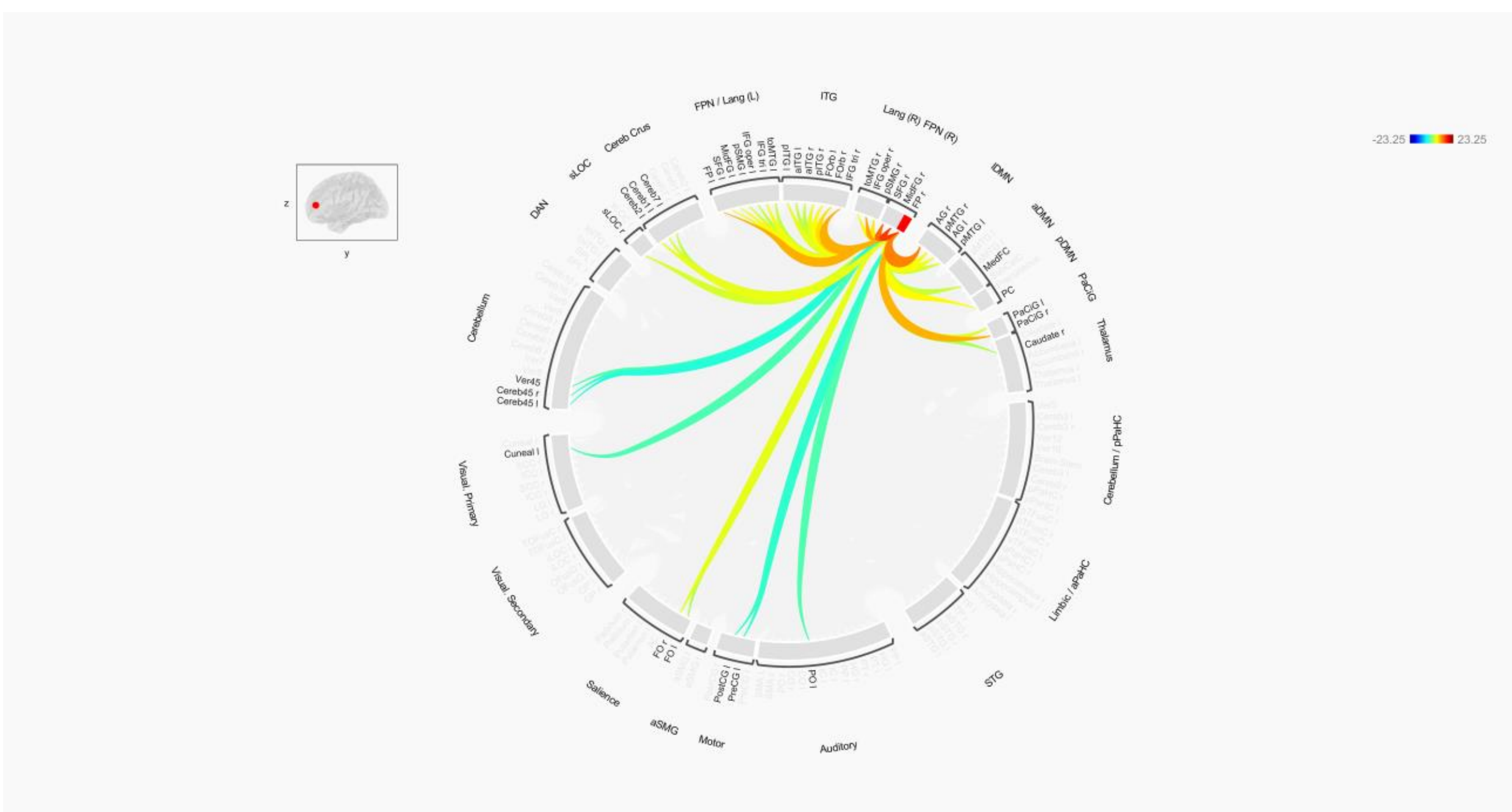

f.) Right Frontal Pole Functional Connectivity (31 h awake, 2:30 pm)

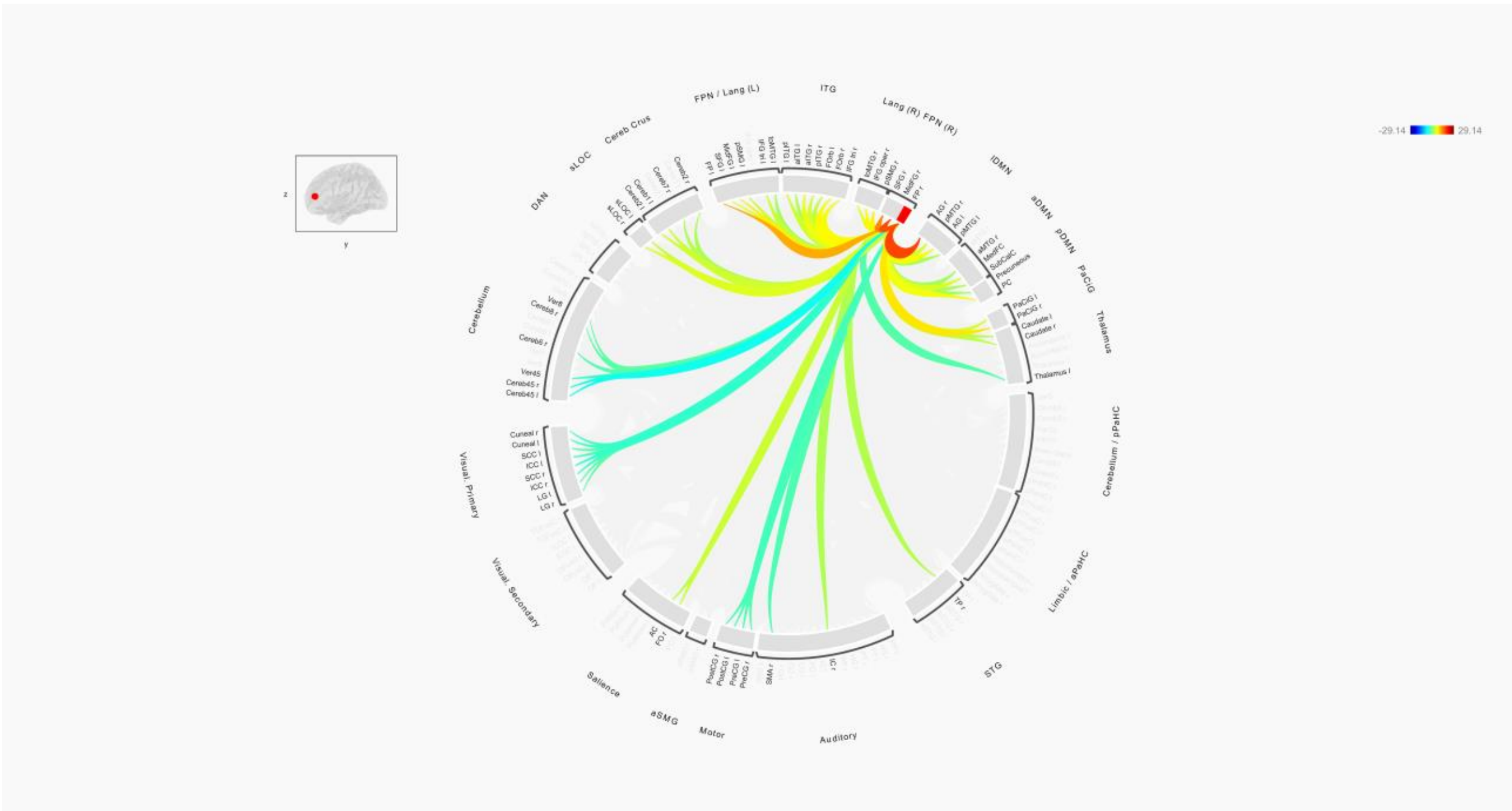

g.) Right Frontal Pole Functional Connectivity (37 h awake, 8:30 pm)

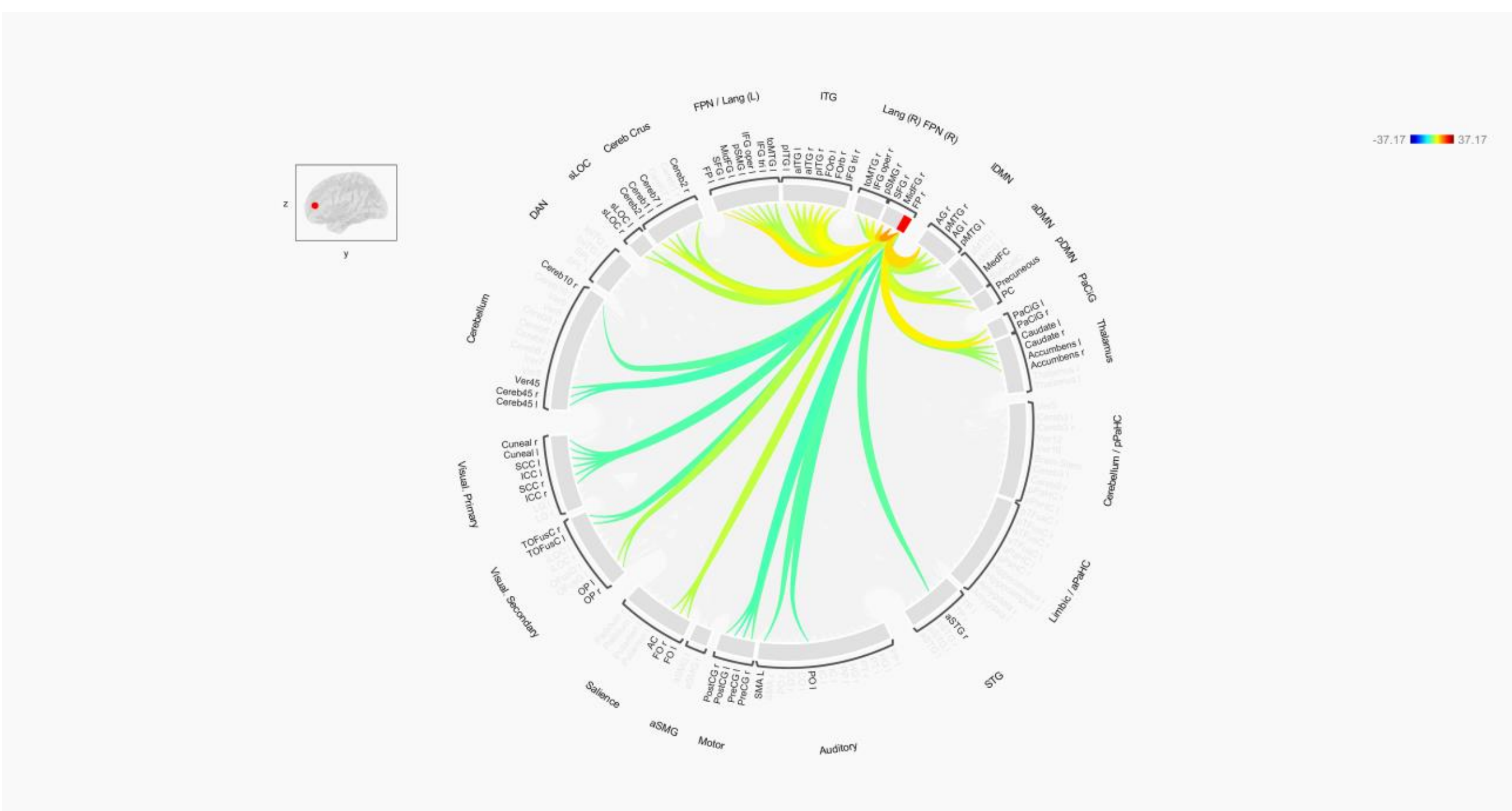

h.) Left Amygdala Functional Connectivity (1 h awake, 8:30 am)

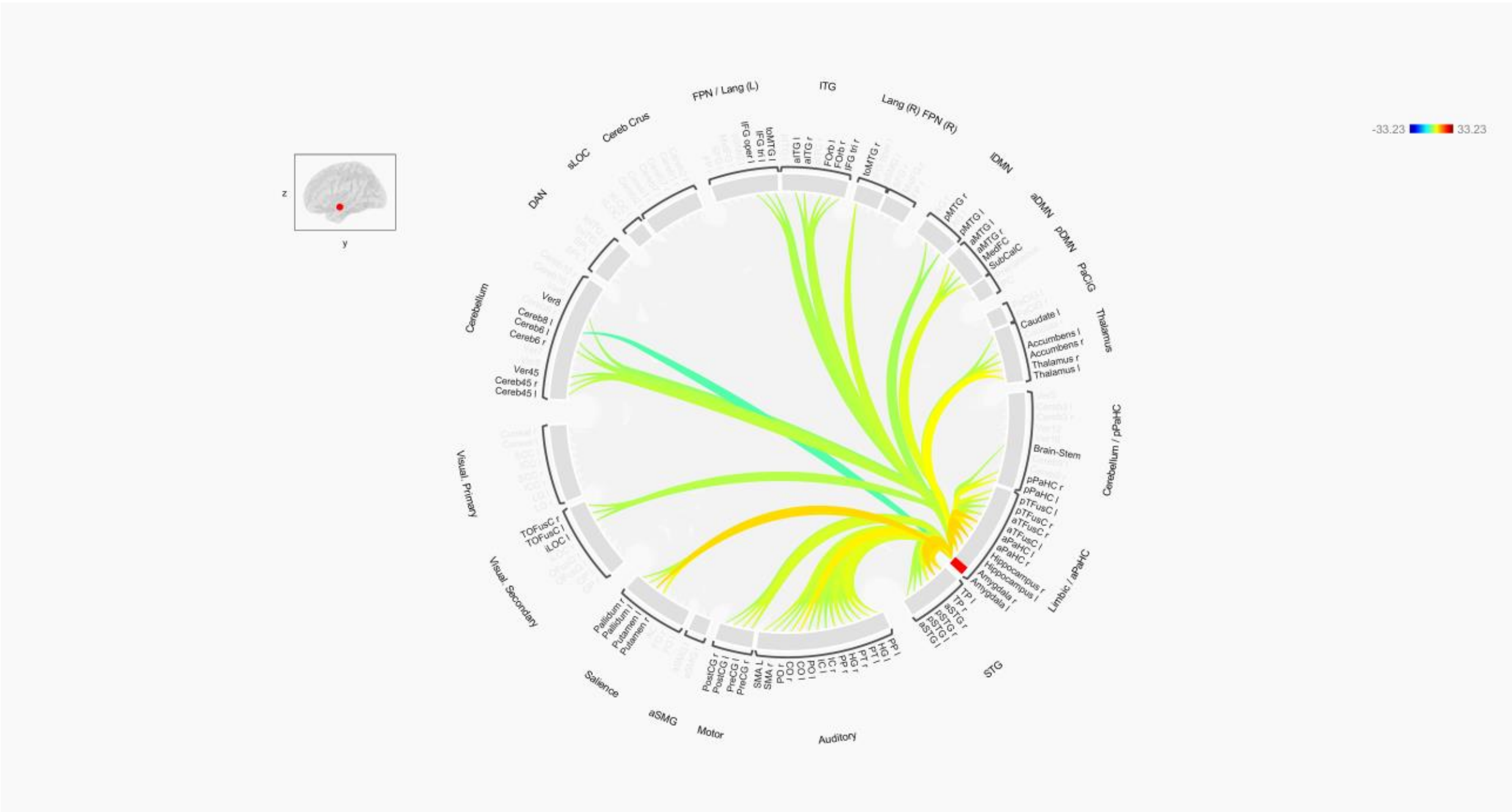

i.) Left Amygdala Functional Connectivity (7 h awake, 2:30 pm)

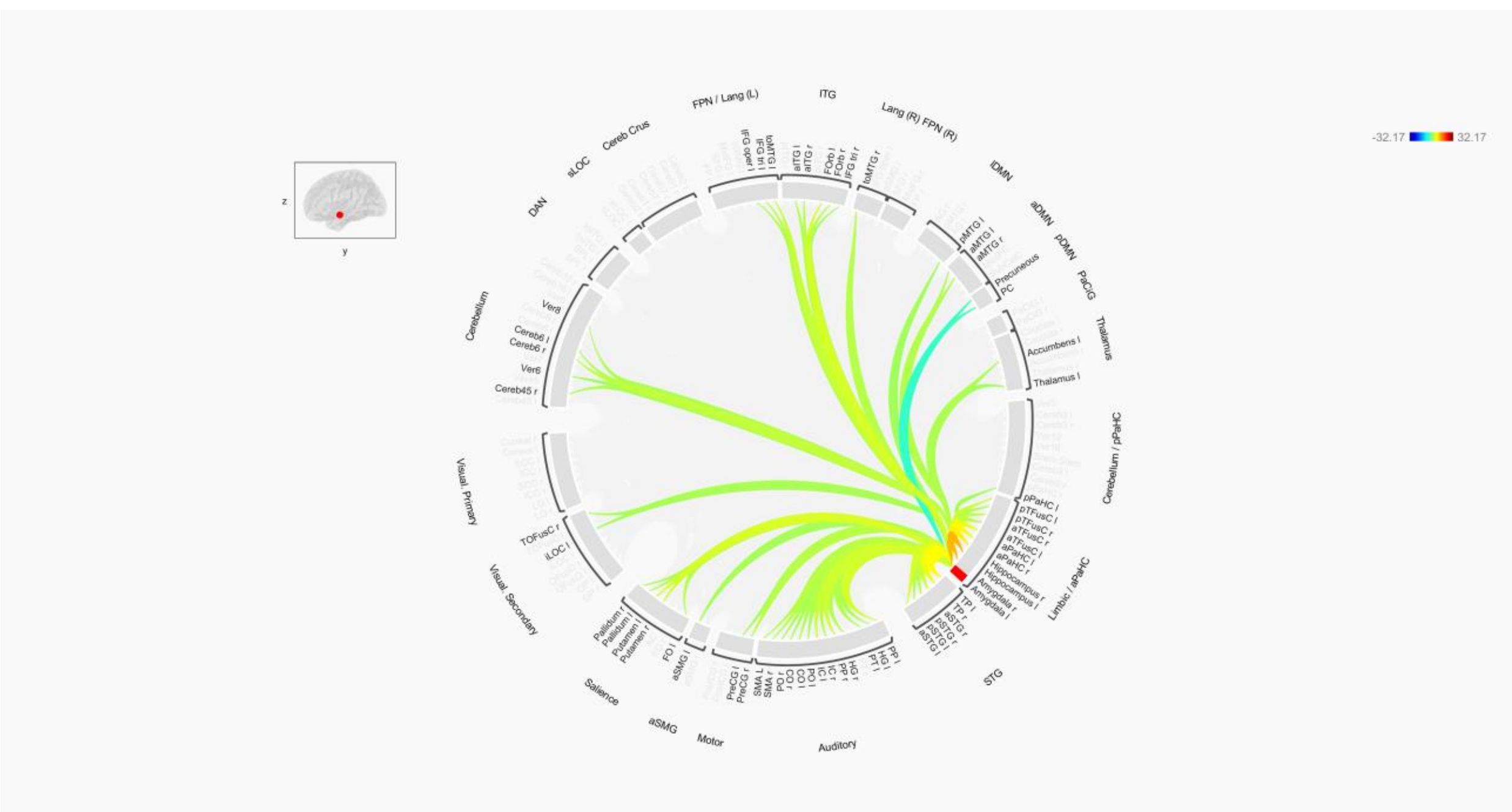

j.) Left Amygdala Functional Connectivity (13 h awake, 8:30 pm)

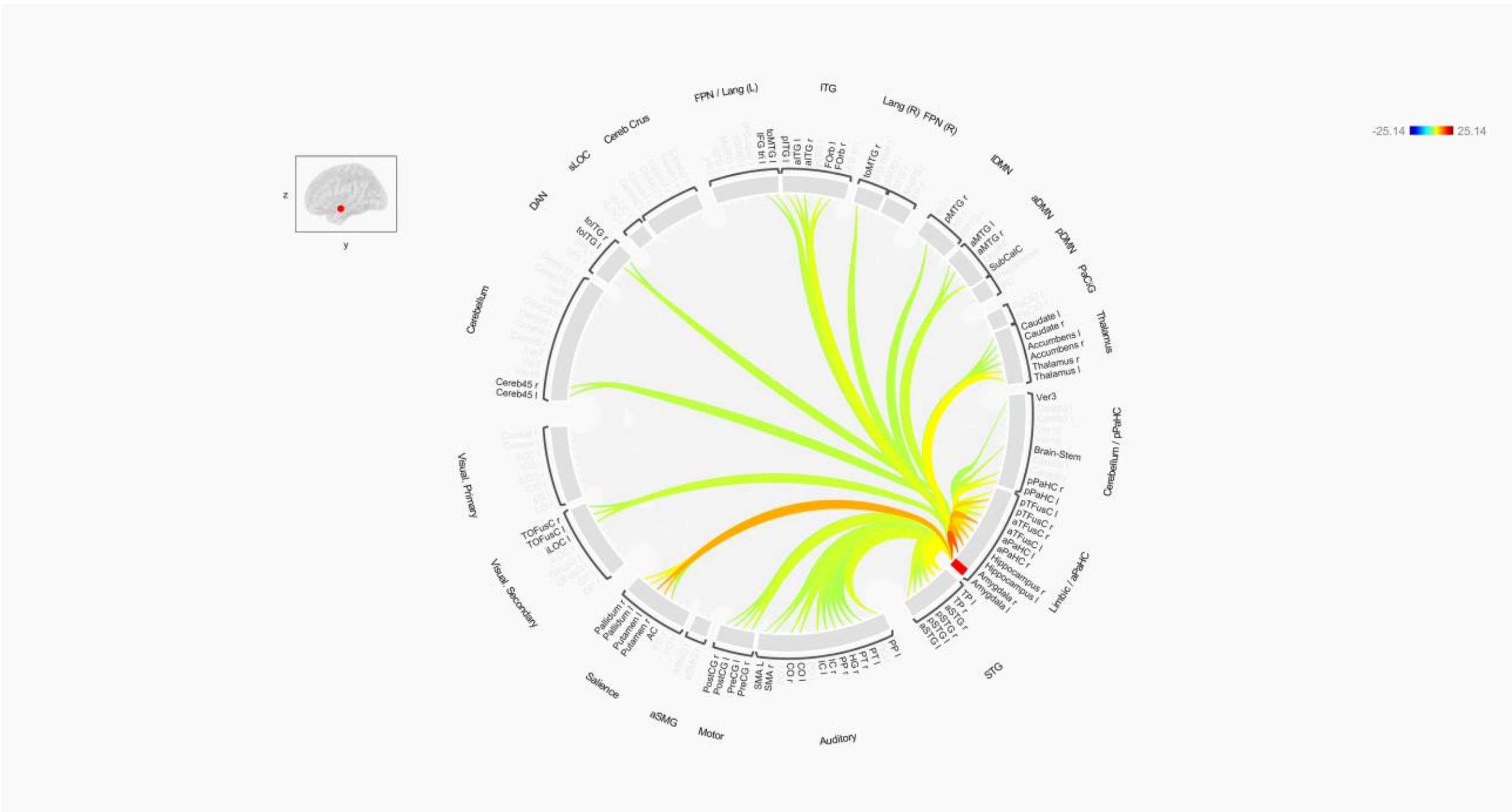

k.) Left Amygdala Functional Connectivity (19 h awake, 2:30 am)

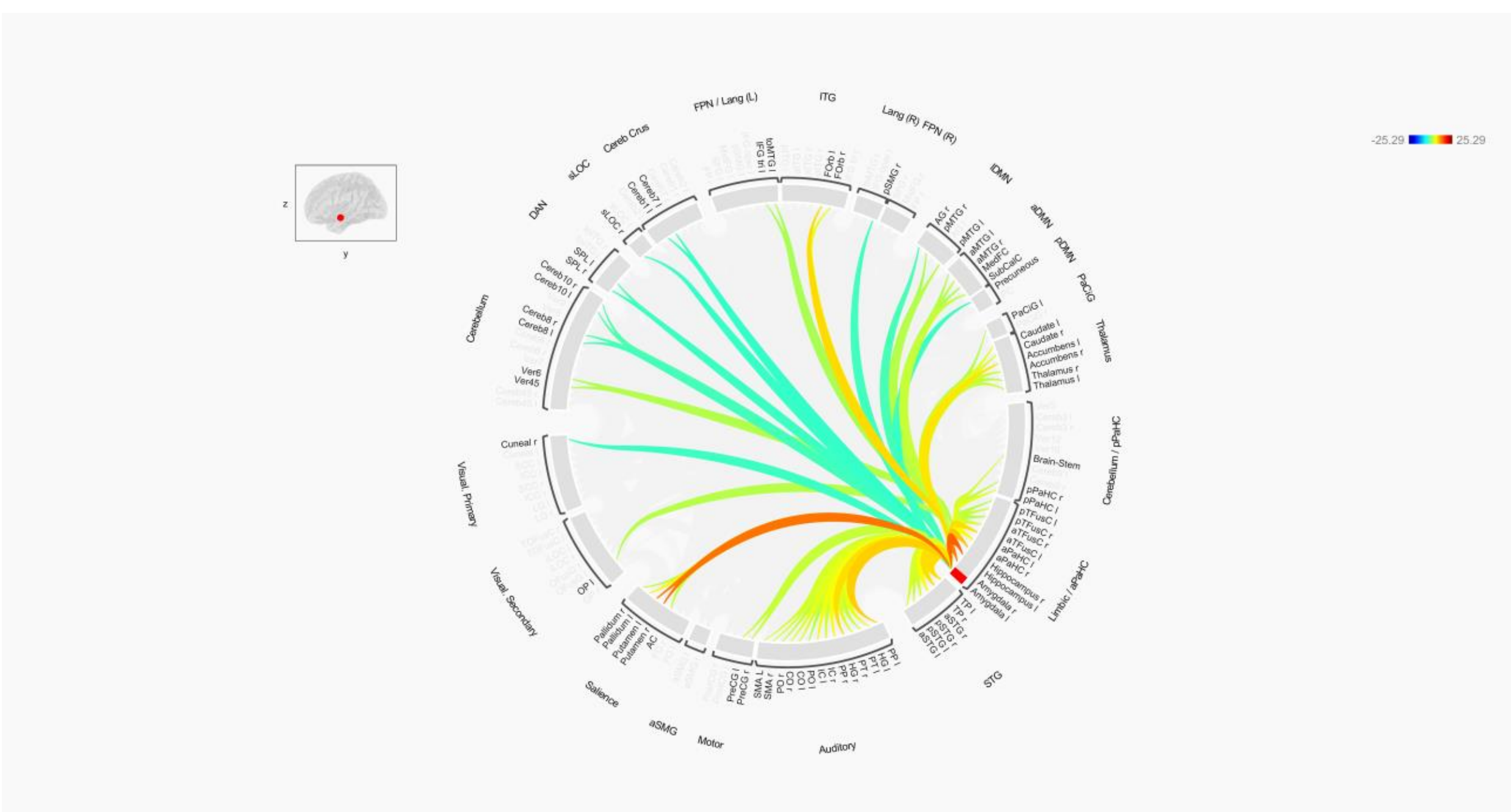

l.) Left Amygdala Functional Connectivity (25 h awake, 8:30 am)

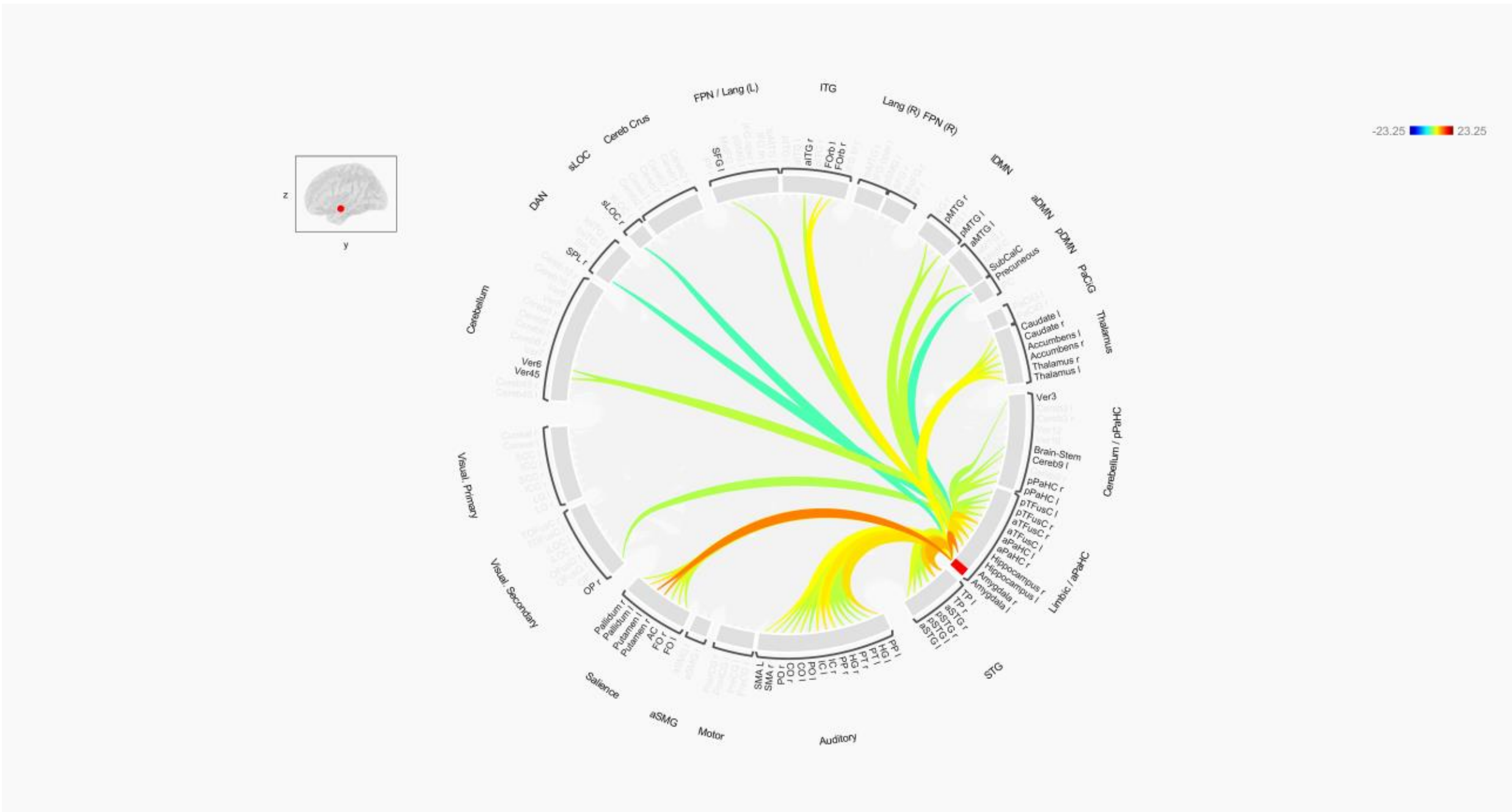

m.) Left Amygdala Functional Connectivity (31 h awake, 2:30 pm)

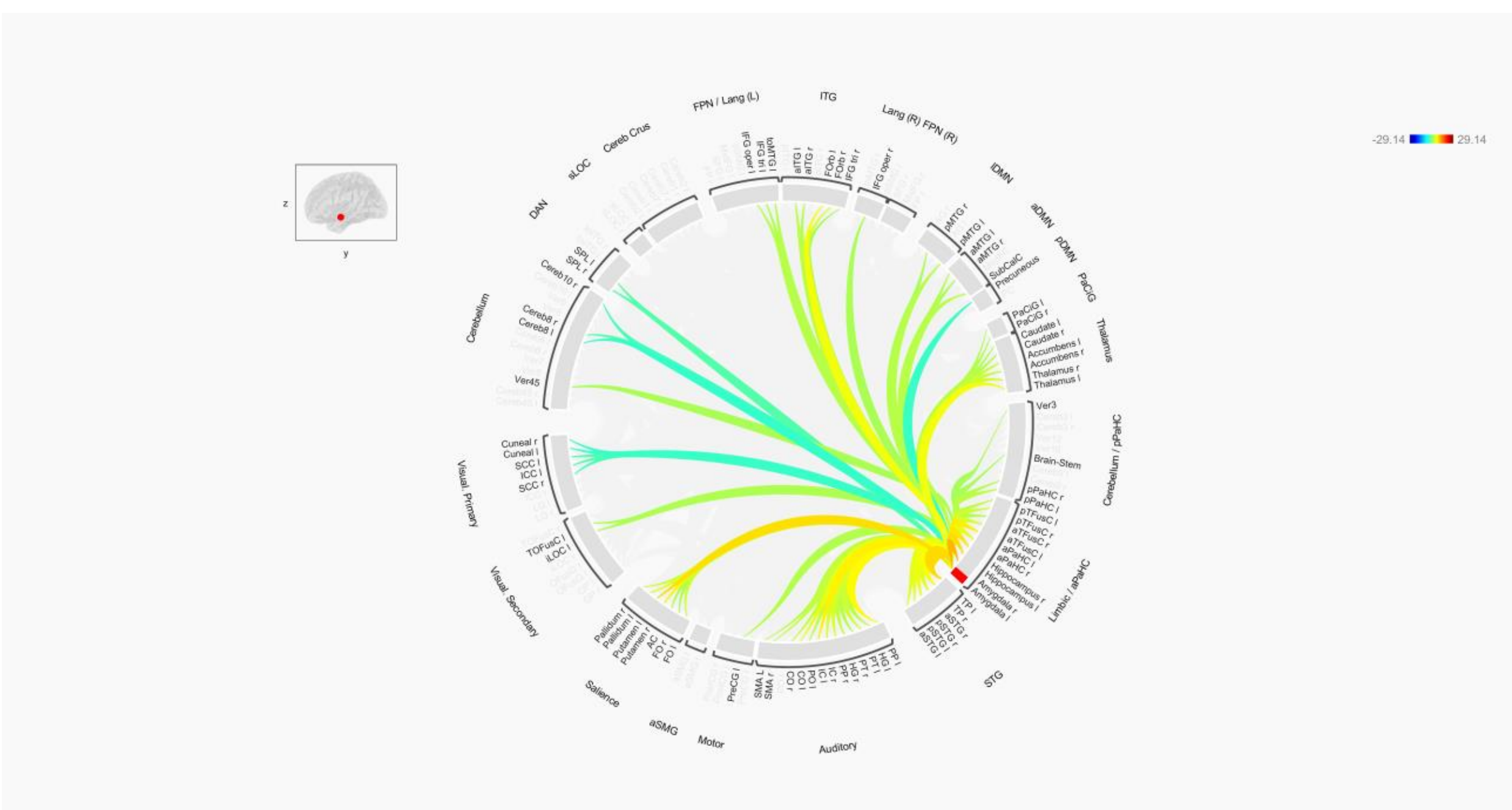

n.) Left Amygdala Functional Connectivity (37 h awake, 8:30 pm)

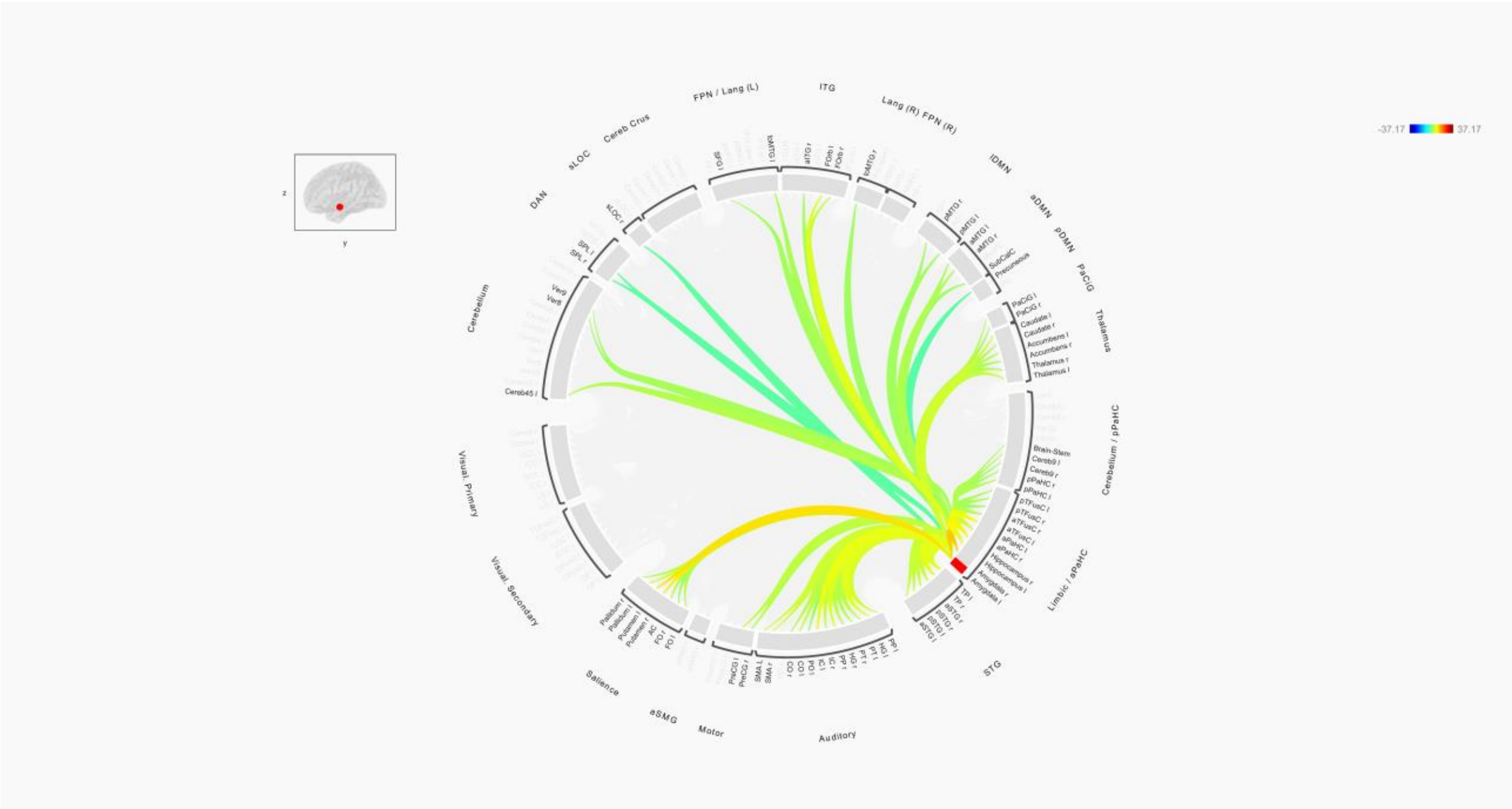

o.) Key Anatomical Abbreviations

Visual. Primary
atlas.Cuneal r (Cuneal Cortex Right)
atlas.Cuneal l (Cuneal Cortex Left)
atlas.SCC l (Supracalcarine Cortex Left)
atlas.ICC l (Intracalcarine Cortex Left)
atlas.SCC r (Supracalcarine Cortex Right)
atlas.ICC r (Intracalcarine Cortex Right)
atlas.LG l (Lingual Gyrus Left)
atlas.LG r (Lingual Gyrus Right)
atlas.TOFusC r (Temporal Occipital Fusiform Cortex Right)
atlas.TOFusC l (Temporal Occipital Fusiform Cortex Left)
atlas.iLOC l (Lateral Occipital Cortex, inferior division Left)
atlas.iLOC r (Lateral Occipital Cortex, inferior division Right)
atlas.OFusG r (Occipital Fusiform Gyrus Right)
atlas.OFusG l (Occipital Fusiform Gyrus Left)
atlas.OP l (Occipital Pole Left)

atlas.OP r (Occipital Pole Right)
atlas.Pallidum r
atlas.Pallidum l
atlas.Putamen l
atlas.Putamen r
atlas.AC (Cingulate Gyrus, anterior division)
atlas.FO r (Frontal Operculum Cortex Right)
atlas.FO l (Frontal Operculum Cortex Left)
atlas.aSMG l (Supramarginal Gyrus, anterior division Left)
atlas.aSMG r (Supramarginal Gyrus, anterior division Right)
atlas.PostCG r (Postcentral Gyrus Right)
atlas.PostCG l (Postcentral Gyrus Left)
atlas.PreCG l (Precentral Gyrus Left)
atlas.PreCG r (Precentral Gyrus Right)
atlas.SMA L(Juxtapositional Lobule Cortex -formerly Supplementary Motor Cortex- Left)
atlas.SMA r (Juxtapositional Lobule Cortex -formerly Supplementary Motor Cortex- Right)
atlas.PO r (Parietal Operculum Cortex Right)
atlas.CO r (Central Opercular Cortex Right)
atlas.CO l (Central Opercular Cortex Left)
atlas.PO l (Parietal Operculum Cortex Left)
atlas.IC l (Insular Cortex Left)
atlas.IC r (Insular Cortex Right)
atlas.PP r (Planum Polare Right)
atlas.HG r (Heschl's Gyrus Right)
atlas.PT r (Planum Temporale Right)
atlas.PT l (Planum Temporale Left)
atlas.HG l (Heschl's Gyrus Left)
atlas.PP l (Planum Polare Left)
atlas.aSTG l (Superior Temporal Gyrus, anterior division Left)
atlas.pSTG l (Superior Temporal Gyrus, posterior division Left)
atlas.pSTG r (Superior Temporal Gyrus, posterior division Right)
atlas.aSTG r (Superior Temporal Gyrus, anterior division Right)
atlas.TP r (Temporal Pole Right)
atlas.TP l (Temporal Pole Left)
atlas.Amygdala l
atlas.Amygdala r
atlas.Hippocampus l
atlas.Hippocampus r
atlas.aPaHC r (Parahippocampal Gyrus, anterior division Right)
atlas.aPaHC l (Parahippocampal Gyrus, anterior division Left)

atlas.aTFusC l (Temporal Fusiform Cortex, anterior division Left)
atlas.aTFusC r (Temporal Fusiform Cortex, anterior division Right)
atlas.pTFusC r (Temporal Fusiform Cortex, posterior division Right)
atlas.pTFusC l (Temporal Fusiform Cortex, posterior division Left)
atlas.pPaHC l (Parahippocampal Gyrus, posterior division Left)
atlas.pPaHC r (Parahippocampal Gyrus, posterior division Right)
atlas.Cereb9 r (Cerebelum 9 Right)
atlas.Cereb9 l (Cerebelum 9 Left)
atlas.Brain-Stem
atlas.Ver10 (Vermis 10)
atlas.Ver12 (Vermis 1 2)
atlas.Cereb3 r (Cerebelum 3 Right)
atlas.Cereb3 l (Cerebelum 3 Left)
atlas.Ver3 (Vermis 3)
atlas.Thalamus l
atlas.Thalamus r
atlas.Accumbens r
atlas.Accumbens l
atlas.Caudate r
atlas.Caudate l
atlas.PaCiG r (Paracingulate Gyrus Right)
atlas.PaCiG l (Paracingulate Gyrus Left)
atlas.PC (Cingulate Gyrus, posterior division)
atlas.Precuneous (Precuneous Cortex)
atlas.SubCalC (Subcallosal Cortex)
atlas.MedFC (Frontal Medial Cortex)
atlas.aMTG r (Middle Temporal Gyrus, anterior division Right)
atlas.aMTG l (Middle Temporal Gyrus, anterior division Left)
atlas.pMTG l (Middle Temporal Gyrus, posterior division Left)
atlas.AG l (Angular Gyrus Left)
atlas.pMTG r (Middle Temporal Gyrus, posterior division Right)
atlas.AG r (Angular Gyrus Right)
atlas.FP r (Frontal Pole Right)
atlas.MidFG r (Middle Frontal Gyrus Right)
atlas.SFG r (Superior Frontal Gyrus Right)
atlas.pSMG r (Supramarginal Gyrus, posterior division Right)
atlas.IFG oper r (Inferior Frontal Gyrus, pars opercularis Right)
atlas.toMTG r (Middle Temporal Gyrus, temporooccipital part Right)
atlas.IFG tri r (Inferior Frontal Gyrus, pars triangularis Right)
atlas.FOrb r (Frontal Orbital Cortex Right)

atlas.FOrb l (Frontal Orbital Cortex Left)
atlas.pITG r (Inferior Temporal Gyrus, posterior division Right)
atlas.aITG r (Inferior Temporal Gyrus, anterior division Right)
atlas.aITG l (Inferior Temporal Gyrus, anterior division Left)
atlas.pITG l (Inferior Temporal Gyrus, posterior division Left)
atlas.toMTG l (Middle Temporal Gyrus, temporooccipital part Left)
atlas.IFG tri l (Inferior Frontal Gyrus, pars triangularis Left)
atlas.IFG oper l (Inferior Frontal Gyrus, pars opercularis Left)
atlas.pSMG l (Supramarginal Gyrus, posterior division Left)
atlas.MidFG l (Middle Frontal Gyrus Left)
atlas.SFG l (Superior Frontal Gyrus Left)
atlas.FP l (Frontal Pole Left)
atlas.Cereb2 r (Cerebelum Crus2 Right)
atlas.Cereb1 r (Cerebelum Crus1 Right)
atlas.Cereb7 r (Cerebelum 7b Right)
atlas.Cereb7 l (Cerebelum 7b Left)
atlas.Cereb1 l (Cerebelum Crus1 Left)
atlas.Cereb2 l (Cerebelum Crus2 Left)
atlas.sLOC l (Lateral Occipital Cortex, superior division Left)
atlas.sLOC r (Lateral Occipital Cortex, superior division Right)
atlas.toITG r (Inferior Temporal Gyrus, temporooccipital part Right)
atlas.toITG l (Inferior Temporal Gyrus, temporooccipital part Left)
atlas.SPL l (Superior Parietal Lobule Left)
atlas.SPL r (Superior Parietal Lobule Right)
atlas.Cereb10 r (Cerebelum 10 Right)
atlas.Cereb10 l (Cerebelum 10 Left)
atlas.Ver9 (Vermis 9)
atlas.Ver8 (Vermis 8)
atlas.Cereb8 r (Cerebelum 8 Right)
atlas.Cereb8 l (Cerebelum 8 Left)
atlas.Cereb6 l (Cerebelum 6 Left)
atlas.Cereb6 r (Cerebelum 6 Right)
atlas.Ver7 (Vermis 7)
atlas.Ver6 (Vermis 6)
atlas.Ver45 (Vermis 4 5)
atlas.Cereb45 r (Cerebelum 4 5 Right)
atlas.Cereb45 l (Cerebelum 4 5 Left)
