# Supplementary material for: Oscillatory network efficiency predicts mood and fatigue during sleep deprivation
Source: Commun Biol. 2026 May 16;9:1041. doi: 10.1038/s42003-026-10250-8 (PMC13438542; doi:10.1038/s42003-026-10250-8)
Supplement: Supplementary file 3 [file 42003_2026_10250_MOESM3_ESM.pdf]

Description of Additional Supplementary files

File name: Supplementary Data 1

Description: This file contains the raw connectivity data underlying the significant regional network changes shown in Figure 4. Values represent Fisher z-transformed correlation coefficients between the seed region and target ROI for each individual at the timepoints corresponding to maximal and minimal regional network efficiency.
