# Supplementary material for: Oscillatory network efficiency predicts mood and fatigue during sleep deprivation
Source: Commun Biol. 2026 May 16;9:1041. doi: 10.1038/s42003-026-10250-8 (PMC13438542; doi:10.1038/s42003-026-10250-8)
Supplement: Supplementary file 4 [file 42003_2026_10250_MOESM5_ESM.pdf]

nature portfolio

Corresponding author(s): David Negelspach, Alisa Huskey, Kathryn Kennedy, Jungwon Cha, Michael Grandner Daniel Forger, William D.S. Killgore

Last updated by author(s): 04/21/2026

Reporting Summary

Nature Portfolio wishes to improve the reproducibility of the work that we publish. This form provides structure for consistency and transparency in reporting. For further information on Nature Portfolio policies, see our Editorial Policies and the Editorial Policy Checklist.

Statistics

For all statistical analyses, confirm that the following items are present in the figure legend, table legend, main text, or Methods section.

n/a	Confirmed	
☐	☒	The exact sample size (*n*) for each experimental group/condition, given as a discrete number and unit of measurement
☐	☒	A statement on whether measurements were taken from distinct samples or whether the same sample was measured repeatedly
☐	☒	The statistical test(s) used AND whether they are one- or two-sided<br>*Only common tests should be described solely by name; describe more complex techniques in the Methods section.*
☐	☒	A description of all covariates tested
☐	☒	A description of any assumptions or corrections, such as tests of normality and adjustment for multiple comparisons
☐	☒	A full description of the statistical parameters including central tendency (e.g. means) or other basic estimates (e.g. regression coefficient) AND variation (e.g. standard deviation) or associated estimates of uncertainty (e.g. confidence intervals)
☐	☒	For null hypothesis testing, the test statistic (e.g. *F*, *t*, *r*) with confidence intervals, effect sizes, degrees of freedom and *P* value noted<br>*Give P values as exact values whenever suitable.*
☒	☐	For Bayesian analysis, information on the choice of priors and Markov chain Monte Carlo settings
☒	☐	For hierarchical and complex designs, identification of the appropriate level for tests and full reporting of outcomes
☐	☒	Estimates of effect sizes (e.g. Cohen's *d*, Pearson's *r*), indicating how they were calculated

Our web collection on statistics for biologists contains articles on many of the points above.

Software and code

Policy information about availability of computer code

Data collection	Software used for the analysis described in the manuscript includes MATLAB, Statistical and Parametric Mapping (SPM for fMRI analysis), Functional Connectivity toolbox (CONN for connectivity and graph theory analysis), R for confirmatory statistical assessment.
Data analysis	MATLAB is version 2023a, SPM is version 12, CONN is version 22.v2407, R is version 4.3.1. All fMRI code used in this analysis is based on peer-reviewed established methodology using the respective programs. Novel Cosinor code was developed in MATLAB using a trust-region reflective algorithm. Documentation for the algorithm used is referenced in the code availability section.

For manuscripts utilizing custom algorithms or software that are central to the research but not yet described in published literature, software must be made available to editors and reviewers. We strongly encourage code deposition in a community repository (e.g. GitHub). See the Nature Portfolio guidelines for submitting code & software for further information.

Data

Policy information about availability of data

All manuscripts must include a data availability statement. This statement should provide the following information, where applicable:

- Accession codes, unique identifiers, or web links for publicly available datasets
- A description of any restrictions on data availability
- For clinical datasets or third party data, please ensure that the statement adheres to our policy

Data may be made available upon reasonable request to the corresponding author, provided the data being shared adheres to participant privacy rights. Source data for graphs and figures are available at the University of Arizona's Research Data Repository.

Research involving human participants, their data, or biological material

Policy information about studies with human participants or human data. See also policy information about sex, gender (identity/presentation), and sexual orientation and race, ethnicity and racism.

Reporting on sex and gender	There were no restrictions on sex or gender for participation in this study. Sex and gender were determined by self-report, and sample demographics are summarized (9 self-reported females; 11 self-reported males). Individual-level sex and gender data cannot be shared without re-consent from participants. Sex was considered in study design to ensure approximately equal numbers of males and females. These variables were not modeled separately in the analysis, as prior work indicates that resilience and vulnerability to sleep deprivation show substantial inter-individual variability that is not cleanly attributable to sex. For this reason, a population-level approach was used to characterize group-level trends in neural oscillatory dynamics rather than stratified analyses. Findings are therefore generalizable across the sample and not specific to any one subgroup.
Reporting on race, ethnicity, or other socially relevant groupings	No socially constructed variables were inferred or collected from study participants. All demographic information collected was self-reported.
Population characteristics	Mean participant age was 23.55 years (SD = 4.65). Self-reported racial/ethnic identities among participants who completed the study included Hispanic, White, Asian, and “Other.” All participants were healthy controls with no reported psychiatric or neurological conditions. Each participant completed a baseline physical examination and neuropsychological assessment to confirm study eligibility.
Recruitment	Recruitment was conducted via fliers and online advertisements.
Ethics oversight	This study was approved by the University of Arizona’s Institutional Review board.

Note that full information on the approval of the study protocol must also be provided in the manuscript.

Field-specific reporting

Please select the one below that is the best fit for your research. If you are not sure, read the appropriate sections before making your selection.

☒ Life sciences ☐ Behavioural & social sciences ☐ Ecological, evolutionary & environmental sciences

For a reference copy of the document with all sections, see nature.com/documents/nr-reporting-summary-flat.pdf

Life sciences study design

All studies must disclose on these points even when the disclosure is negative.

Sample size	Sample size was initially determined according to funding constraints. Post-hoc power analyses were conducted based on previously published scientific work to confirm sufficient power to detect functional properties specific to sleep deprivation.
Data exclusions	One participant's data was excluded due to the scanner computer crashing.
Replication	Multiple steps were implemented to ensure the reproducibility and reliability of all experimental findings. First, all preprocessing and analytic pipelines were performed using fully scripted workflows in fMRI analysis ensuring that every step can be re-run identically. All analyses were performed using standardized toolboxes with documented software versions. Internal replication was conducted by corroborating different modalities of functional measures; seed-to-voxel analyses mirror changes in structure that are consistent with changes in global efficiency.
Randomization	Randomization was not required for this study design. Functional data analysis included multiple covariate control procedures, as detailed in the neuroimaging methods section. Briefly, preprocessing accounted for motion artifacts, physiological noise, magnetic field distortions, and individual differences in brain morphology.
Blinding	Blinding was not required because the study involved objective neuroimaging across sleep deprivation in healthy participants, rather than variable interventions that could be influenced by experimenter or participant expectations.

Reporting for specific materials, systems and methods

We require information from authors about some types of materials, experimental systems and methods used in many studies. Here, indicate whether each material, system or method listed is relevant to your study. If you are not sure if a list item applies to your research, read the appropriate section before selecting a response.

Materials & experimental systems

n/a	Involved in the study
☒	☐ Antibodies
☒	☐ Eukaryotic cell lines
☒	☐ Palaeontology and archaeology
☒	☐ Animals and other organisms
☒	☐ Clinical data
☒	☐ Dual use research of concern
☒	☐ Plants

Methods

n/a	Involved in the study
☒	☐ ChIP-seq
☒	☐ Flow cytometry
☐	☒ MRI-based neuroimaging

Plants

Seed stocks

Report on the source of all seed stocks or other plant material used. If applicable, state the seed stock centre and catalogue number. If plant specimens were collected from the field, describe the collection location, date and sampling procedures.

Novel plant genotypes

Describe the methods by which all novel plant genotypes were produced. This includes those generated by transgenic approaches, gene editing, chemical/radiation-based mutagenesis and hybridization. For transgenic lines, describe the transformation method, the number of independent lines analyzed and the generation upon which experiments were performed. For gene-edited lines, describe the editor used, the endogenous sequence targeted for editing, the targeting guide RNA sequence (if applicable) and how the editor was applied.

Authentication

Describe any authentication procedures for each seed stock used or novel genotype generated. Describe any experiments used to assess the effect of a mutation and, where applicable, how potential secondary effects (e.g. second site T-DNA insertions, mosiacism, off-target gene editing) were examined.

Magnetic resonance imaging

Experimental design

Design type

Resting-state

Design specifications

Seven resting-state scans were conducted across sleep deprivation for each individual. Scans were taken six-hours apart. Each resting state scan lasted 6 minutes and 12 seconds.

Behavioral performance measures

Behavioral measures included psychomotor vigilance tests (reaction time and lapses), visual analogue mood scale (mean and standard deviation of emotional components), and visual analogue of fatigue scale (mean and standard deviation of fatigue), which were taken at regular intervals throughout the sleep deprivation protocol.

Acquisition

Imaging type(s)

Functional echo planar imaging and structural Magnetization-Prepared Rapid Acquisition with Gradient Echo (MPRAGE)

Field strength

3T

Sequence & imaging parameters

Functional images were acquired using a T2*- weighted echo-planar imaging (EPI) sequence. The imaging parameters were as follows: TR = 2000 ms, TE = 36.0 ms, flip angle = 90°, slice thickness = 2.0 mm, and voxel size = $2.0mm^3$. A total of 60 slices were acquired in an interleaved order, with a field of view (FoV) of 240 mm. The phase encoding direction was A >> P.

Area of acquisition

Whole brain acquired

Diffusion MRI ☐ Used ☒ Not used

Preprocessing

Preprocessing software

MATLAB is version 2023a, SPM is version 12, CONN is version 22.v2407. Processing included realignment, movement and and magnetic distortion correction, slice timing correction, outlier detection, functional segmentation and normalization, structural segmentation and normalization, functional smoothing with Gaussian kernel full-width half maximum = 8mm.

Normalization	Data was normalized using the SPM12's unified segmentation and normalization procedure which is based on non-linear deformation fields (affine regularization) applied both to the structural and functional data.
Normalization template	Normalization space template was based on the Montreal Neurological Institute (M.N.I.)
Noise and artifact removal	Outlier scans were identified using the Artifact Detection Tools (ART) as volumes exhibiting frame-wise displacement > 0.9 mm or global BOLD signal changes exceeding 5 standard deviations. Physiological and other noise sources were controlled using CompCor denoising, which extracts principal components from white matter and cerebrospinal fluid masks to serve as nuisance regressors in the analysis. Functional data were further band-pass filtered to reduce the influence of low-frequency drift and high-frequency physiological signals such as respiration and cardiac activity.
Volume censoring	ART controls for error volumes as defined by global BOLD signal changes exceeding 5 standard deviations

Statistical modeling & inference

Model type and settings	For seed-to-voxel and ROI analyses, connectivity is calculated based on BOLD signal time-series between the seed and target regions, with additional regressors for CompCor components, motion parameters, and ART-identified outlier volumes, using a weighted general linear model. Second-level analyses use mixed-effects models that account for within-subject repeated measures. Multivariate parametric statistics with random effects are applied to voxel-specific connectivity measures. Cluster-level hypothesis tests are then performed using parametric statistics based on Gaussian random field theory.
Effect(s) tested	No stimulus was applied. During the scan, subjects maintained a fixed gaze. A within-subject design was applied to graph-theory measures of regional network global efficiency across the course of sleep deprivation.

Specify type of analysis: ☐ Whole brain ☒ ROI-based ☐ Both

Anatomical location(s)	Regions were based on the Harvard-Oxford atlas (RRID:SCR_001476) and the Automated Anatomical Labeling atlas.
Statistic type for inference (See Eklund et al. 2016)	Statistical thresholds were applied to the individual voxel at p<.001 and to clusters p<.05 after both false discovery rate and family wise error cluster correction.
Correction	All reported regions were significant for both family wise error correction and false discovery rate correction

Models & analysis

n/a	Involved in the study
☐	☒ Functional and/or effective connectivity
☐	☒ Graph analysis
☒	☐ Multivariate modeling or predictive analysis

Functional and/or effective connectivity	Functional connectivity strength was represented by Fisher-transformed bivariate pearson correlation coefficients from a weighted GLM.
Graph analysis	Global efficiency was the primary graph-theoretic measure and was calculated for each participant based on their individual functional connectivity networks. This measure captures the efficiency of information transfer across the functional network and reflects its overall topological organization.
